# Supplementary material for: Multi-dimensional genomic analysis of myoepithelial carcinoma identifies prevalent oncogenic gene fusions
Source: Nat Commun. 2017 Oct 30;8:1197. doi: 10.1038/s41467-017-01178-z (PMC5662567; doi:10.1038/s41467-017-01178-z)
Supplement: Supplementary file 1 — Supplementary Information [file 41467_2017_1178_MOESM1_ESM.pdf]

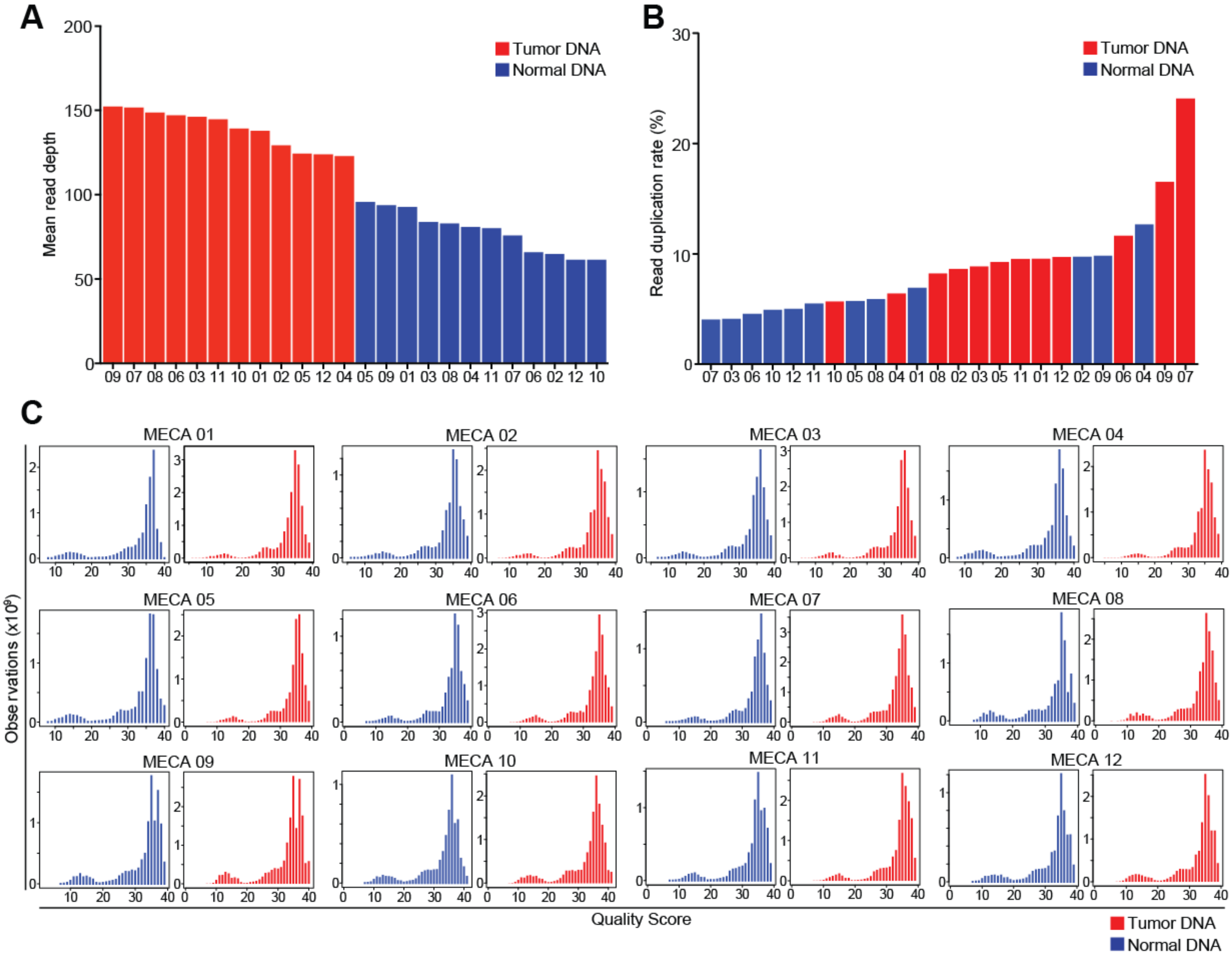

Supplementary Figure 1. Coverage and quality of whole-exome sequencing analysis in cohort 1. (A) Mean read depth, (B) read duplication rate, and (C) read quality score distribution, in tumor and normal DNA of the 12 cases.

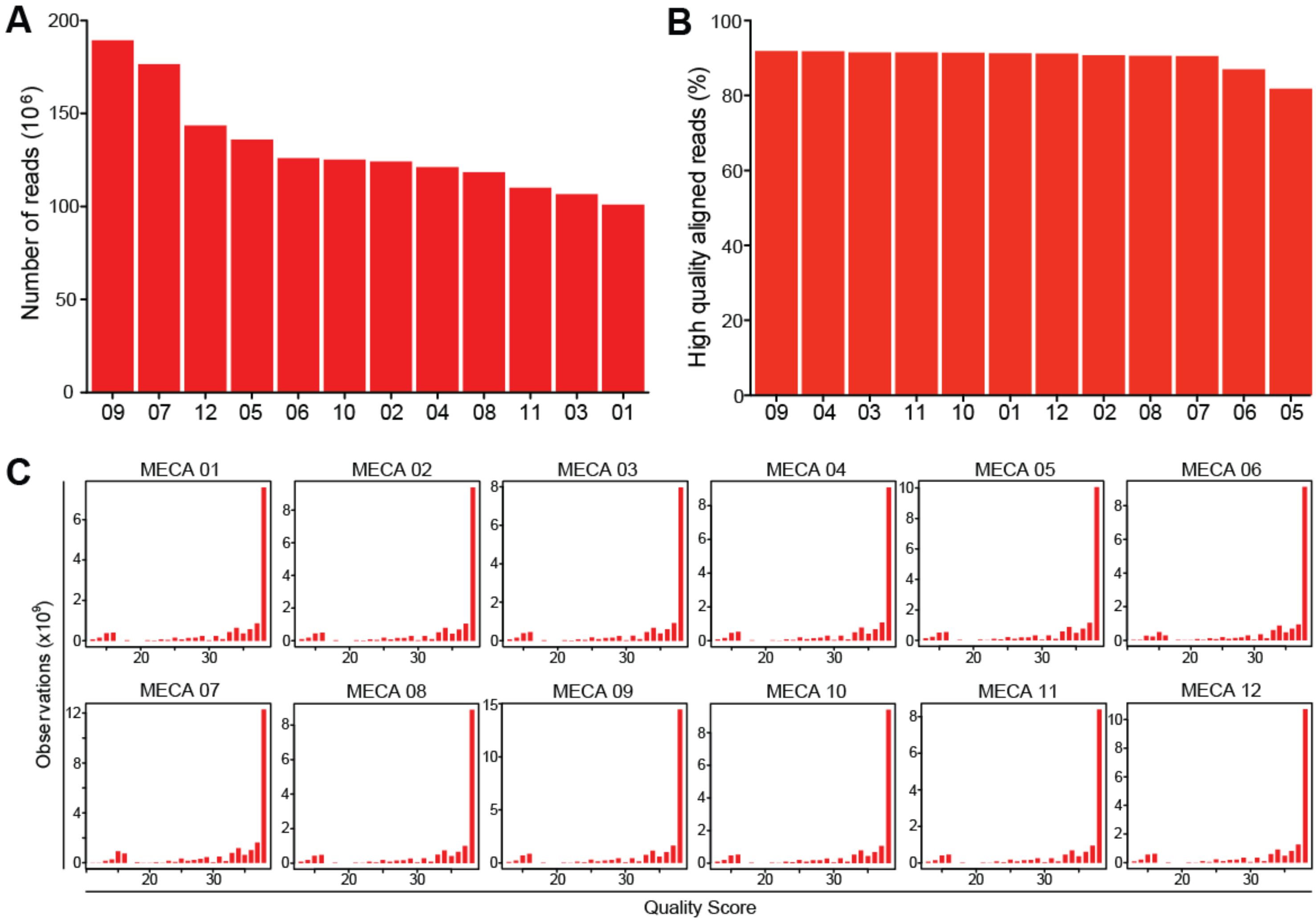

Supplementary Figure 2. Depth and quality of RNA sequencing analysis in cohort 1. (A) Number of reads that passed Illumina's filter. (B) Percent of the reads passing filter that were aligned to the mapping sequence with a mapping quality of Q20 or higher, leading to a <1% probability that the alignment is wrong. (C) Read quality score distribution for each case.

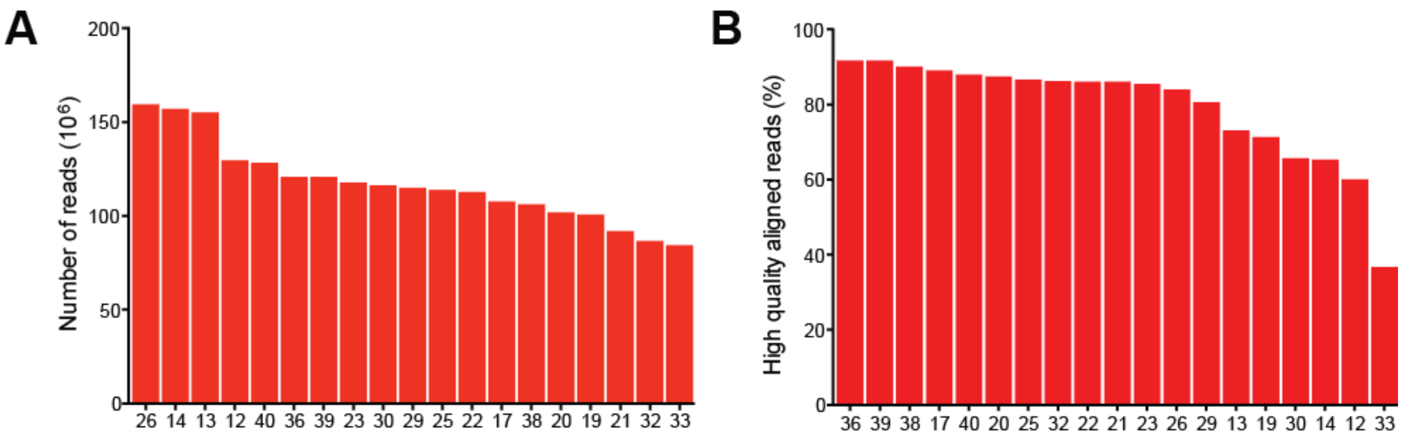

Supplementary Figure 3. Depth and quality of RNA sequencing analysis in cohort 2. (A) Number of reads that passed Illumina's filter. (B) Percent of the reads passing filter that were aligned to the mapping sequence with a mapping quality of Q20 or higher, leading to a <1% probability that the alignment is wrong.

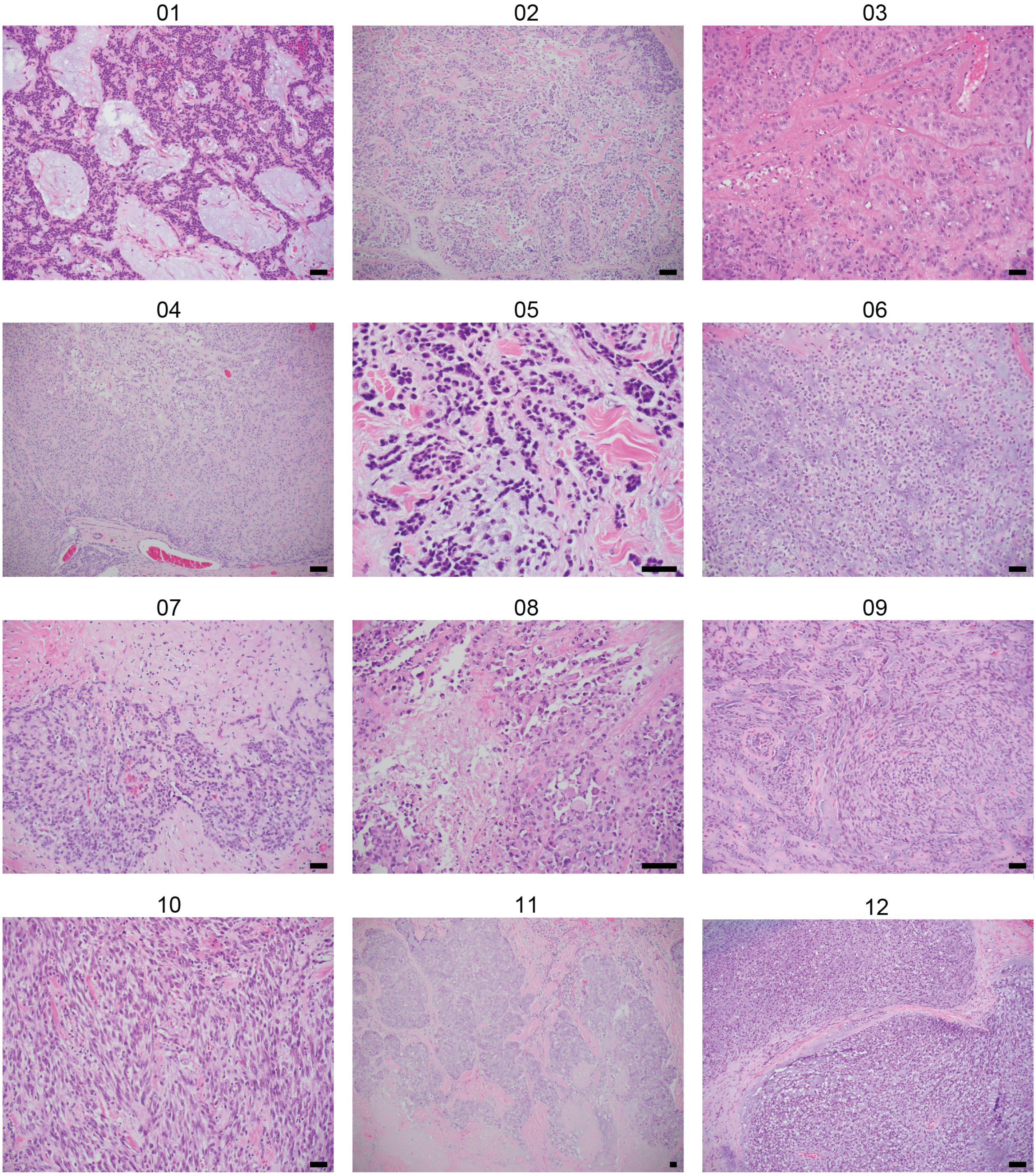

Supplementary Figure 4. H/E staining of MECA01-MECA12. Scale bars, 100 μm.

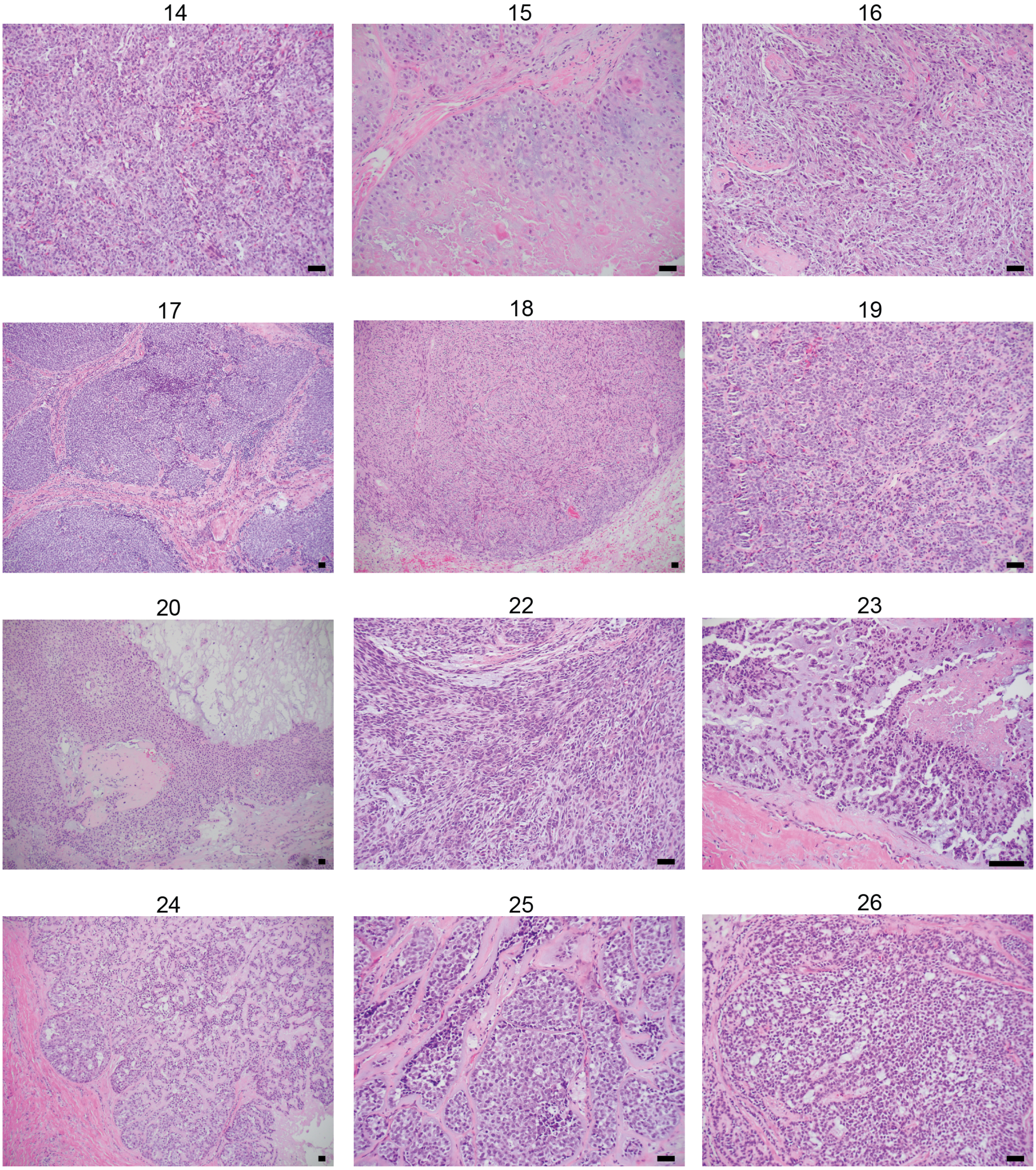

Supplementary Figure 5. H/E staining of MECA14-MECA20 and MECA22-MECA26. Scale bars, 100 μm.

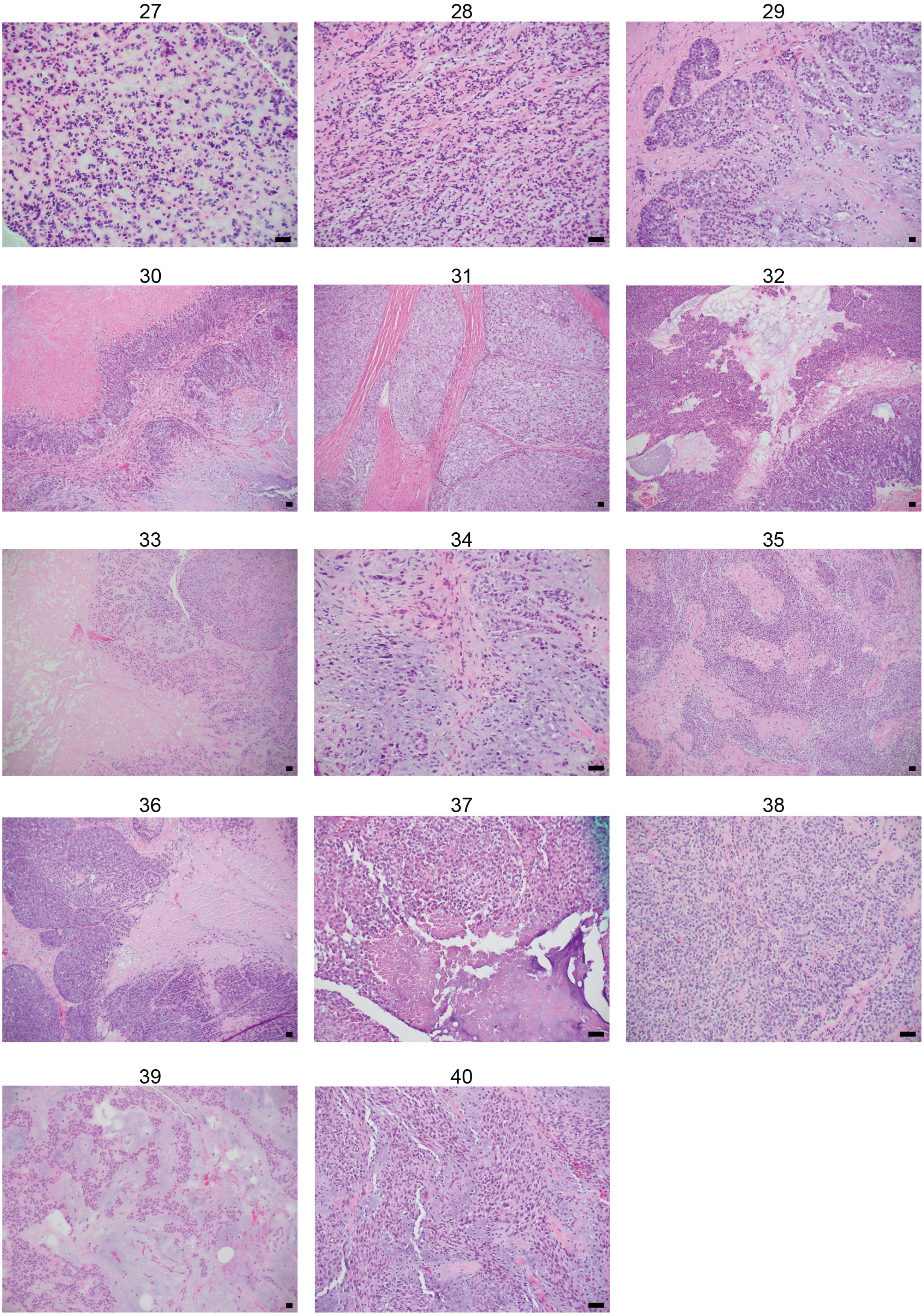

Supplementary Figure 6. H/E staining of MECA27-MECA40. Scale bars, 100 μm.

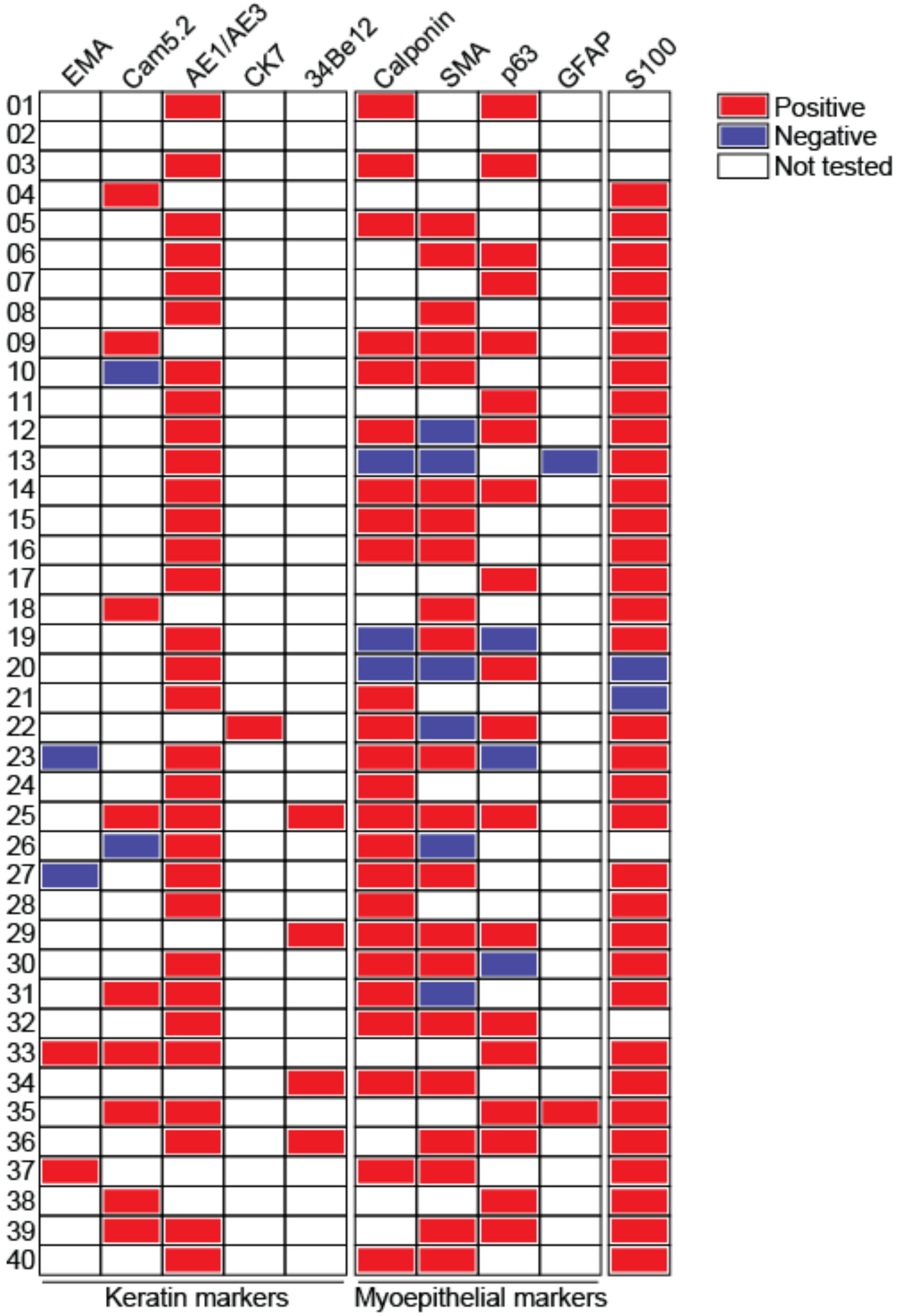

Supplementary Figure 7. Diagnostic IHC analyses. MECAs are positive for at least one keratin marker, and at least one myoepithelial marker or S100. MECA02 was not available for IHC but showed typical MECA histology.

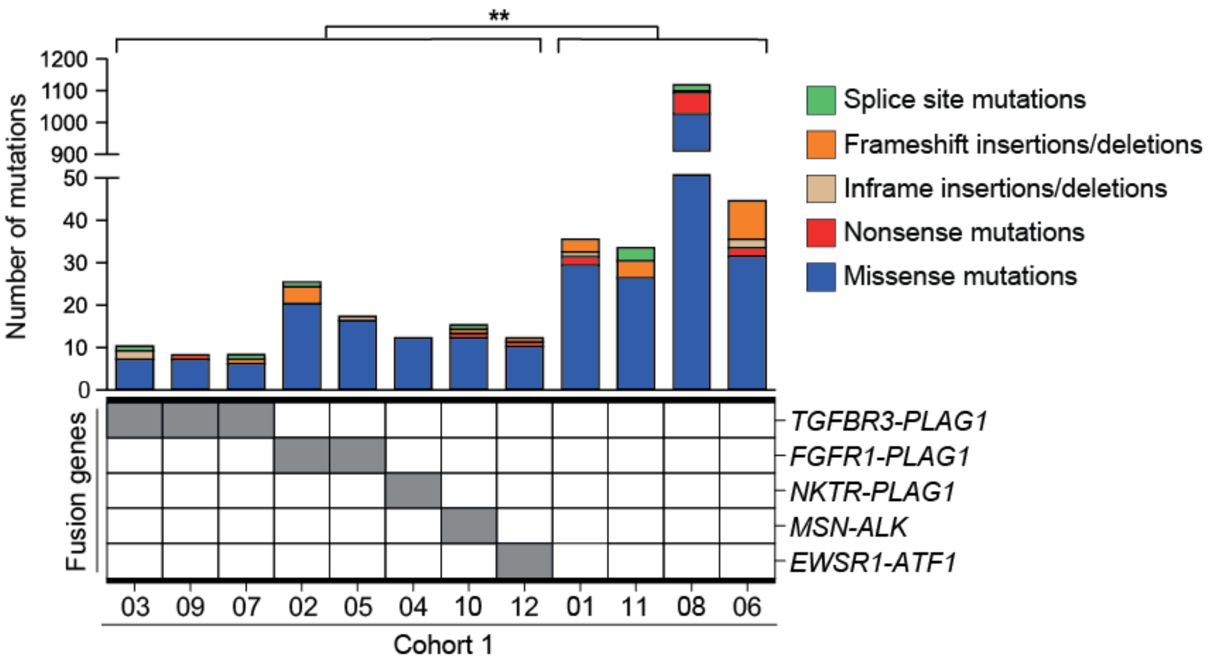

Supplementary Figure 8. Mutation spectrum in cohort 1. $^{**}P < 0.01$ (number of mutations in fusion gene positive versus –negative cases); Mann-Whitney test.

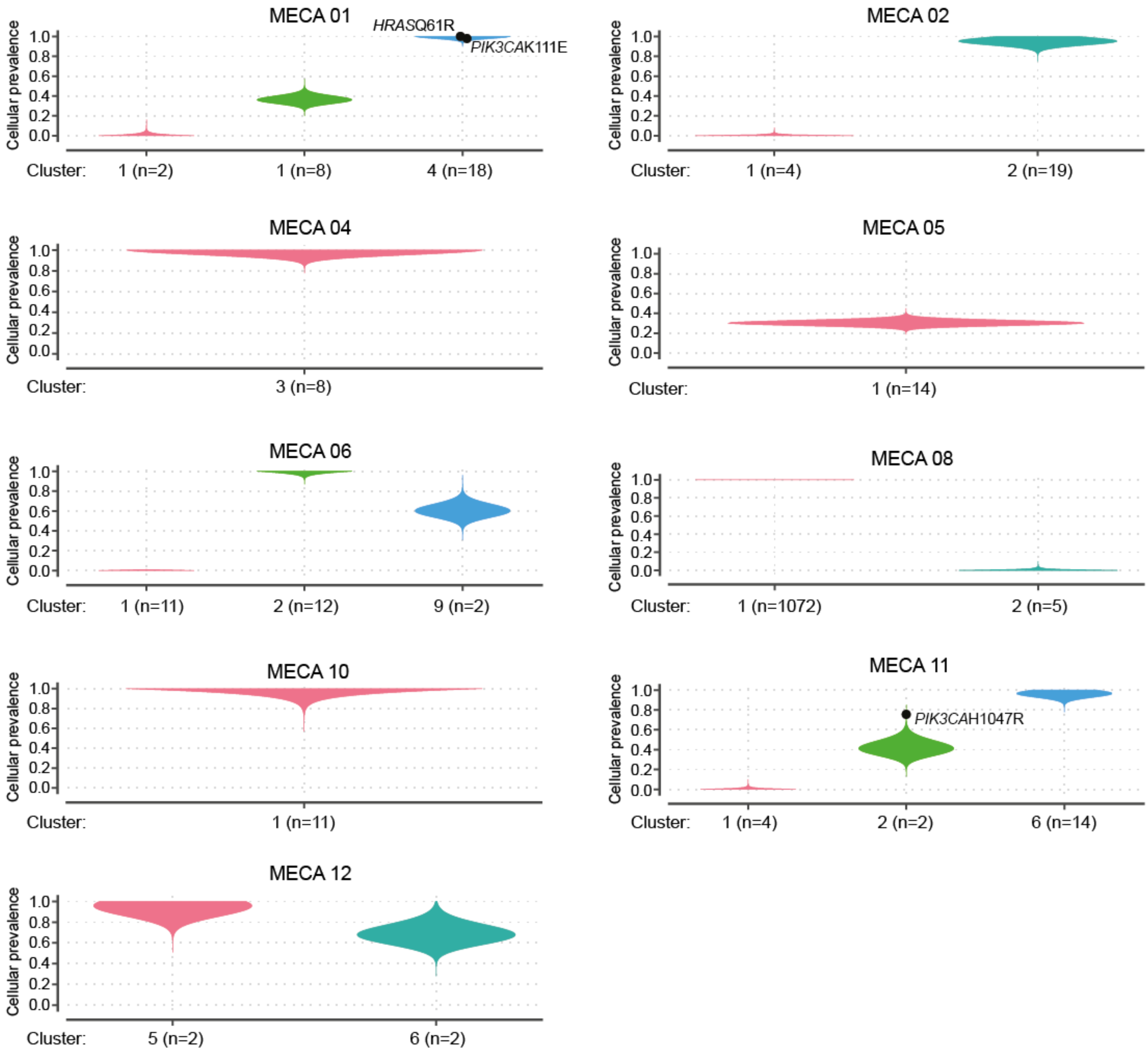

Supplementary Figure 9. Analysis of intratumor mutational heterogeneity in cohort 1. With clustering of cancer cellular prevalence by PyClone (see Methods). Two tumors had less than 10 mutations and were therefore excluded from the analysis. n, number of mutations per cluster.

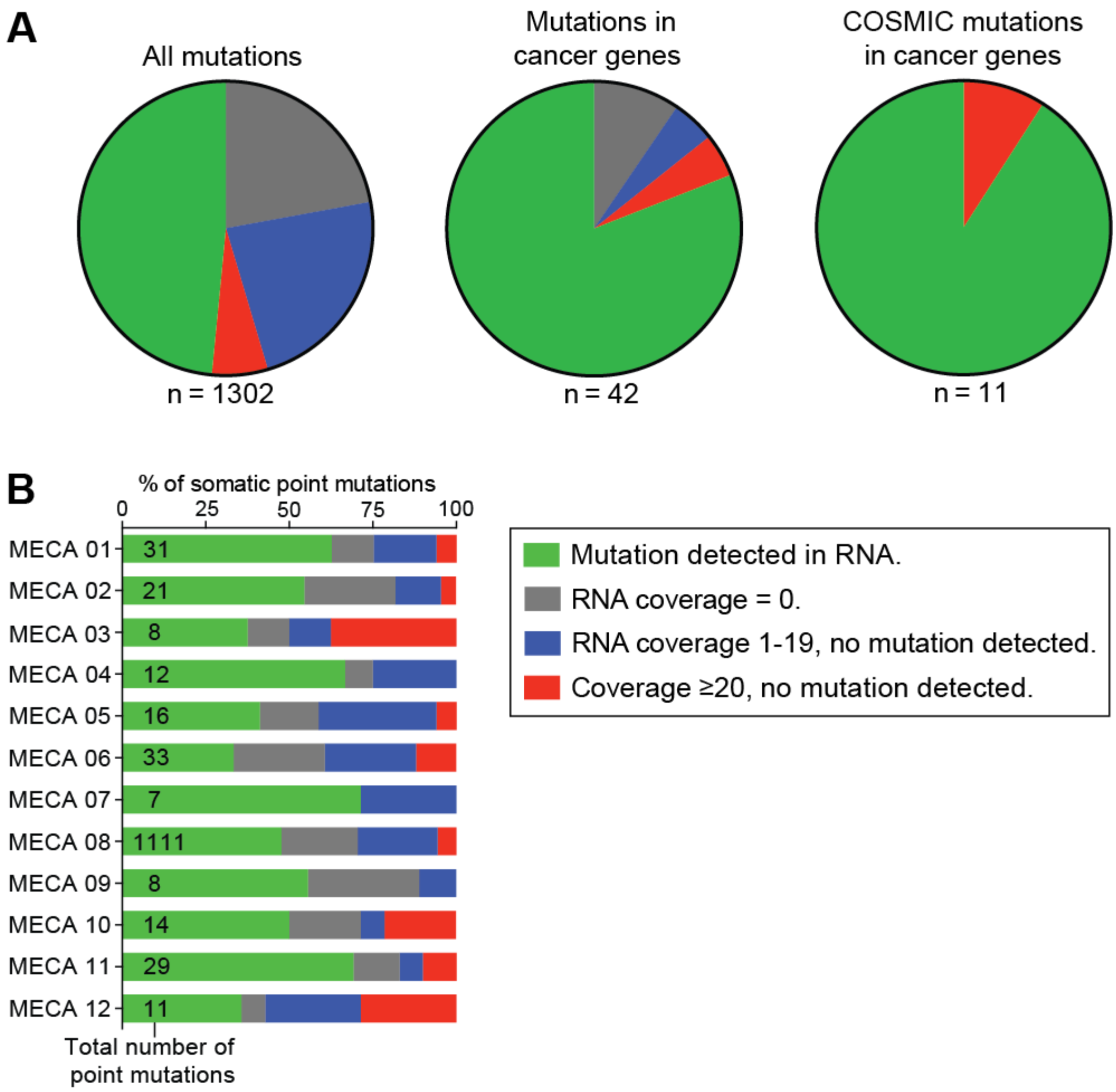

Supplementary Figure 10. Detection of somatic mutations in cohort 1 using RNA sequencing. (A) Pie charts showing all (left), cancer gene mutations only (center) or COSMIC mutations in cancer genes only (right). *Cancer genes are defined by the COSMIC cancer Gene Census list (cancer.sanger.ac.uk/census). (B) Number of somatic point mutations and percent of mutations detected by RNA sequencing for each individual case.

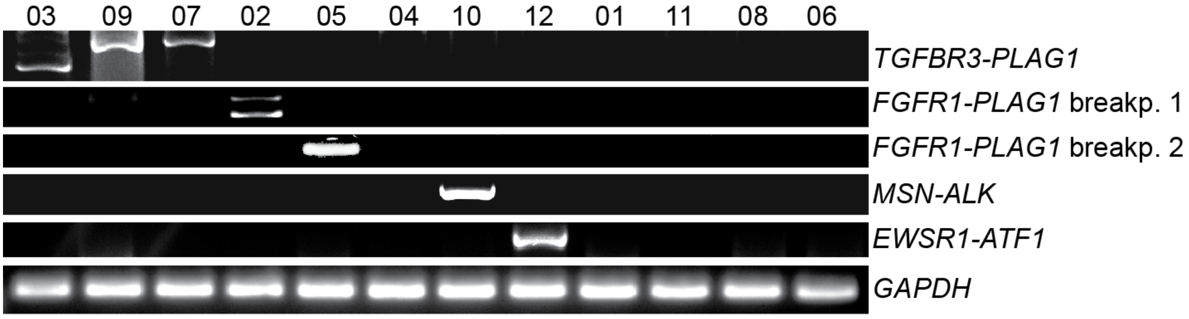

Supplementary Figure 11. Confirmation of fusion genes in cohort 1 using RT-PCR. For primer sequences, see Supplementary Table 1.

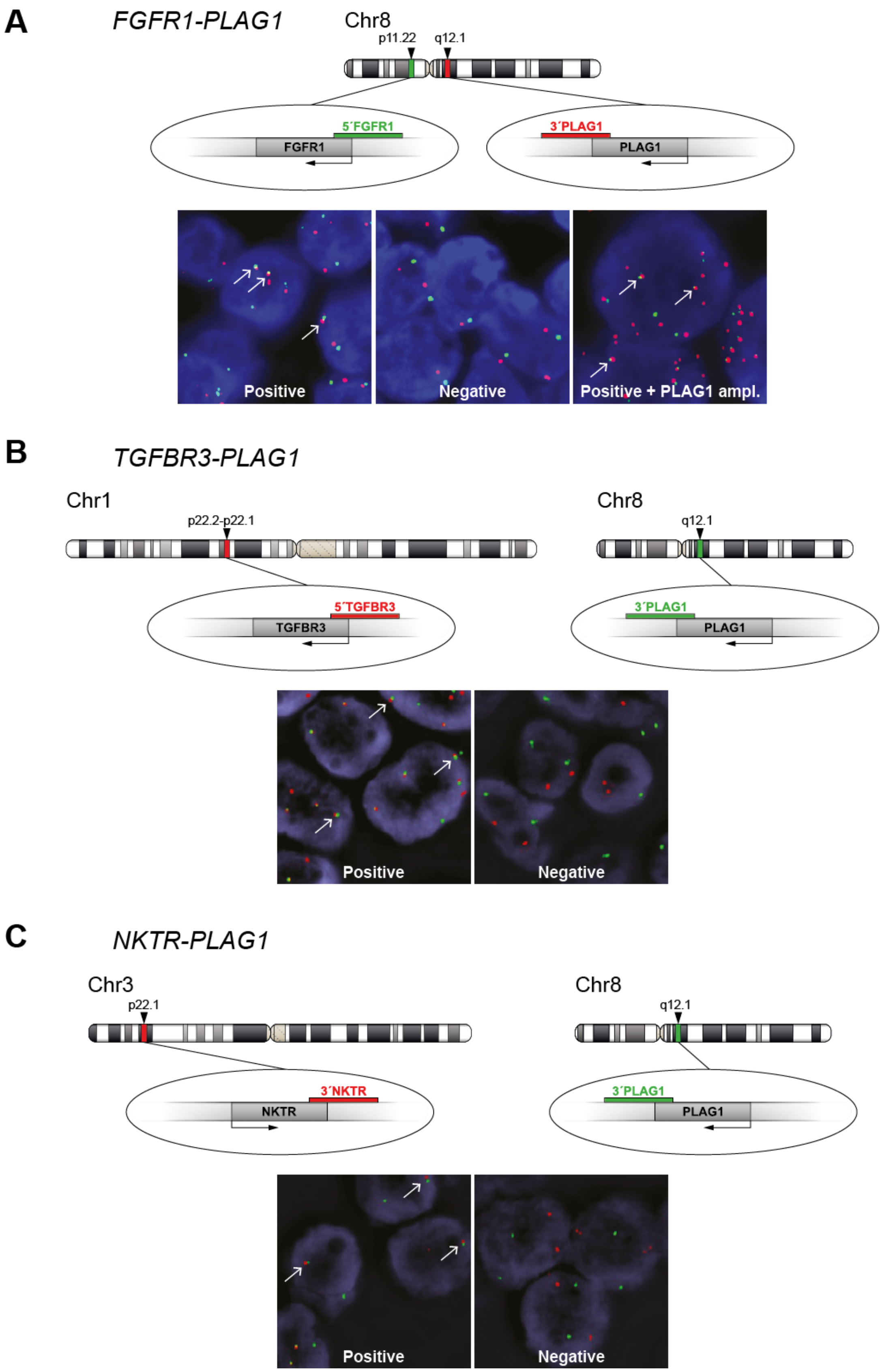

Supplementary Figure 12. Confirmation of *PLAG1* fusion genes in cohort 1 using FISH. White arrows show co-localization of the two probes, consistent with fusion gene formation.

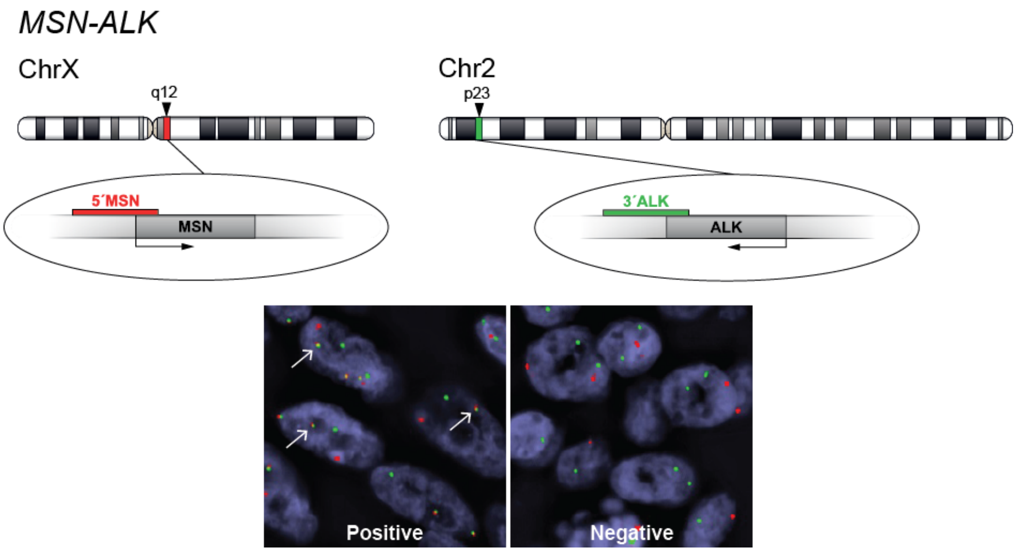

Supplementary Figure 13. Confirmation of the *MSN-ALK* fusion gene using FISH. White arrows show co-localization of the two probes, suggesting fusion gene formation.

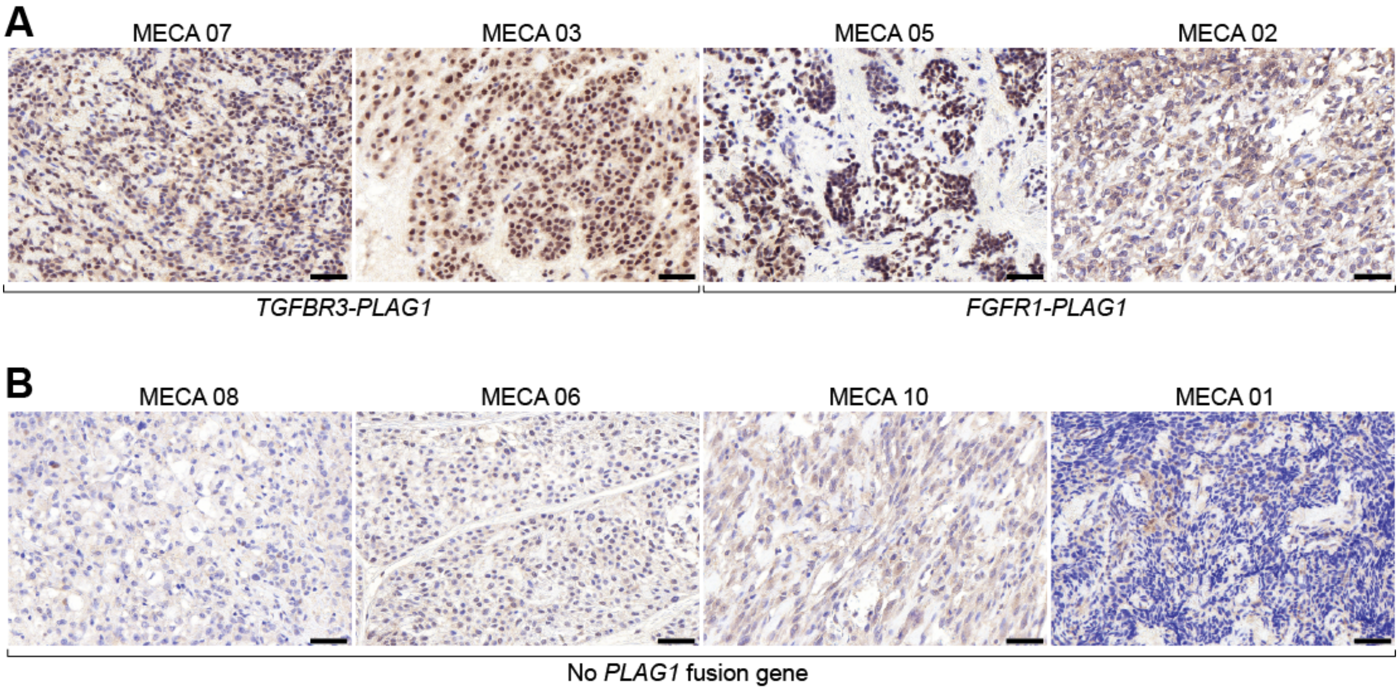

Supplementary Figure 14. Nuclear accumulation of PLAG1 protein in tumors harboring *PLAG1* fusion genes. Immunohistochemistry showing (A) 4 tumors with *TGFBR3-PLAG1* or *NKTR-PLAG1* fusion genes, and (B) 4 randomly selected tumors negative for *PLAG1* fusion genes. Scale bars, 50 µm.

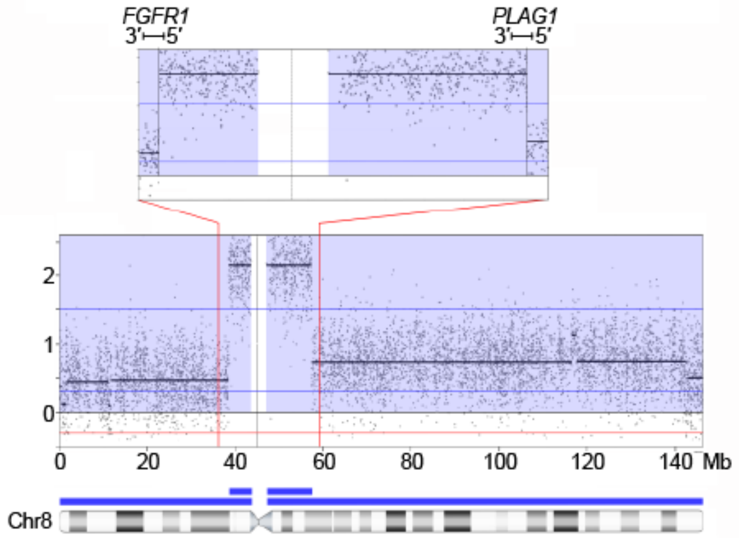

Supplementary Figure 15. Ring chromosome 8 (r8) formation in an *FGFR1-PLAG1* positive case of MECA ex-PA. Array-CGH data showing amplification of the centromeric portion of chromosome 8 with breakpoints in *PLAG1* and *FGFR1*, consistent with r8 formation.

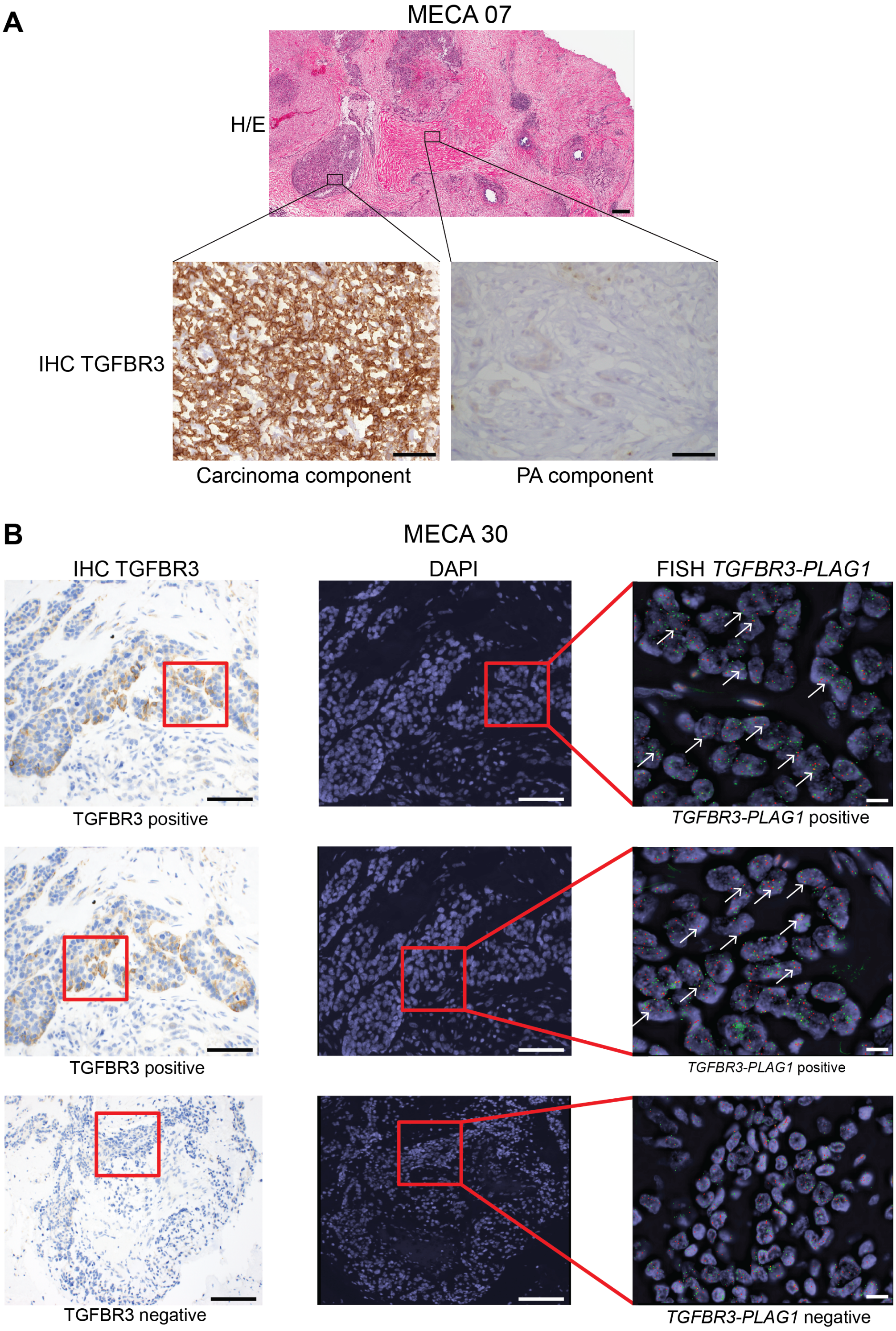

Supplementary Figure 16. Focally positive TGFBR3 staining in MECA ex-PA cases positive for the *TGFBR3-PLAG1* fusion gene. (A) Case 07; TGFBR3 staining is positive in the carcinoma and negative in the PA component. Scale bars, 1 mm (top) and 100 µm (bottom). (B) Case 30; co-localization of the *TGFBR3-PLAG1* fusion detected by FISH and positive TGFBR3 by IHC. Scale bars, 100 µm (left and middle panel) and 10 µm (right panel).

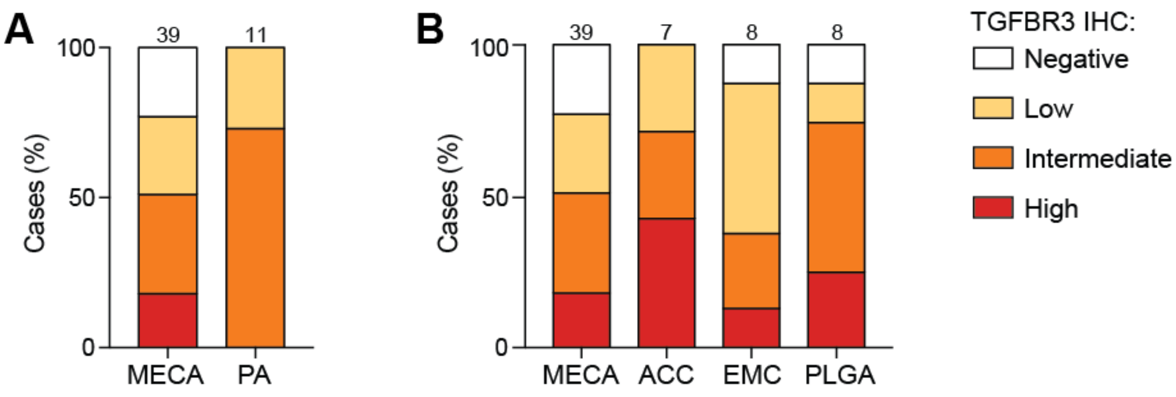

Supplementary Figure 17. Quantification of TGFBR3 IHC analysis in different types of salivary gland tumors. Number of analyzed cases for each tumor type is stated on top of bars. (A) MECA versus PA. (B) MECA versus other salivary gland cancers. ACC, adenoid cystic carcinoma; EMC, epithelial myoepithelial carcinoma; PLGA, polymorphic low-grade adenocarcinoma. *P* (high TGFBR3) = n.s. Fisher's exact test.

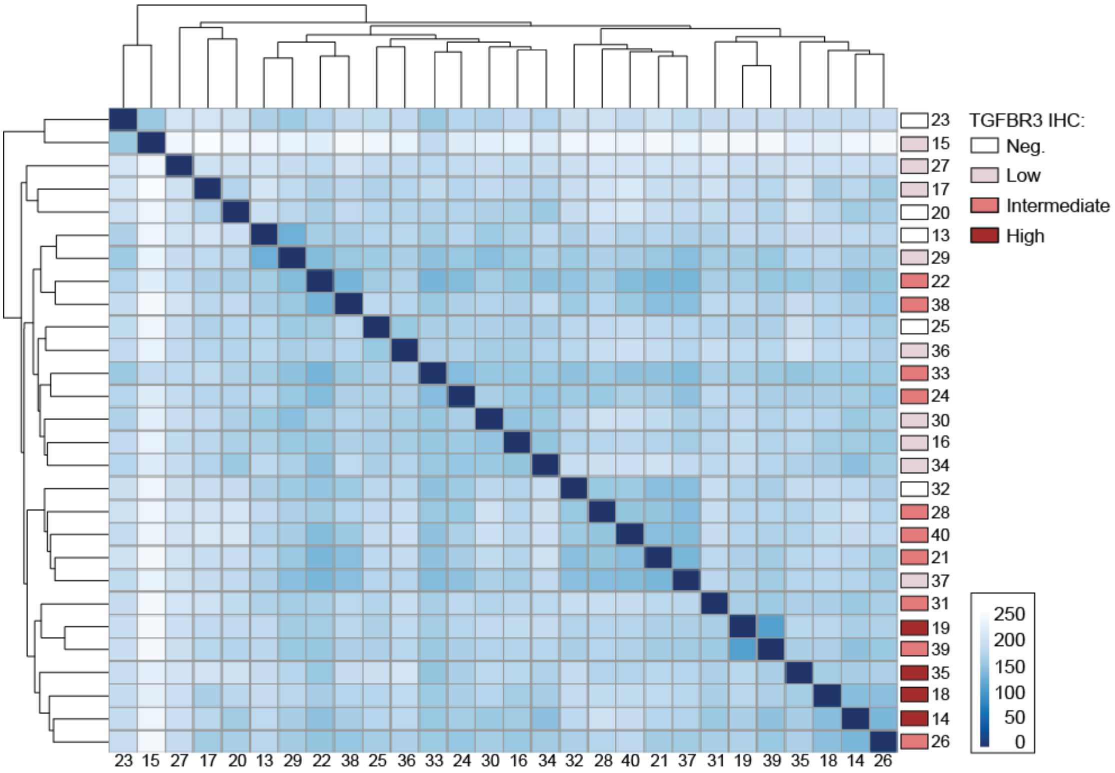

Supplementary Figure 18. Poisson sample clustering based on gene expression in cohort 2. Annotated by TGFBR3 IHC results.

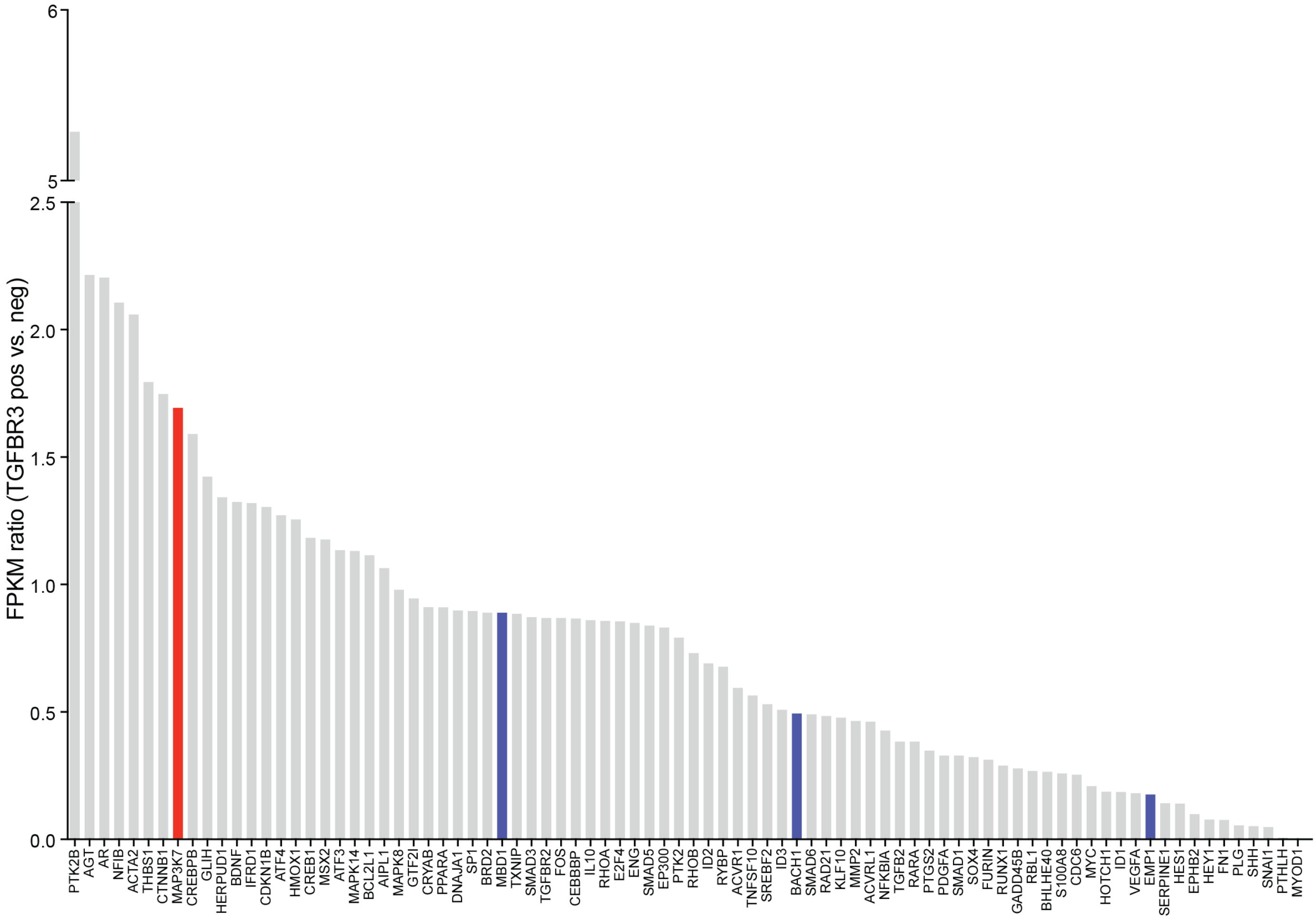

Supplementary Figure 19. Effect of TGFBR3 overexpression on TGF-ß signaling in MECA. Expression of 84 TGF-ß pathway effector genes[1], shown as ratio between tumors with high (n = 4) versus those with negative, low or intermediate (n = 8) TGFBR3 IHC levels. Red and blue bars denote genes significantly up- and downregulated in TGFBR3 high tumors, respectively (Student's t test, $P < 0.05$). However, the expression of these genes was not significantly different between the groups when adjusting for multiple comparisons. The tumors in cohort 2 were excluded from this analysis, since FFPE preservation may affect mRNA levels.

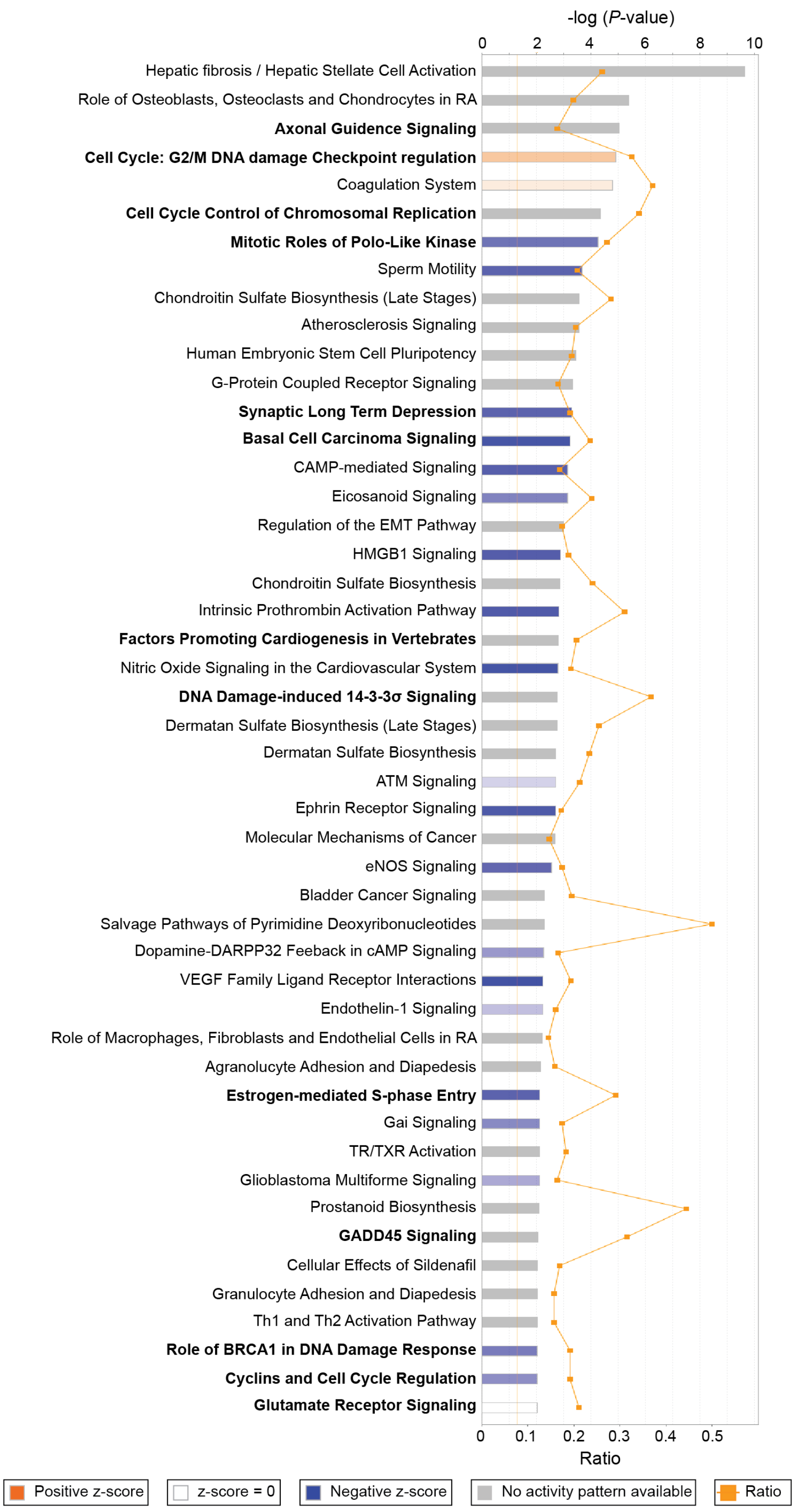

Supplementary Figure 20. Effect of TGFBR3 overexpression on signaling pathways in MECA. Ingenuity pathway analysis[1] of cohort 1 tumors with high (n = 4) versus those with negative, low or intermediate (n = 8) TGFBR3 IHC levels. Pathways with significantly different activity between the groups (*P* <0.01) are shown, ranked by *P*-value. Pathways in bold were also significantly different (*P* <0.01) in TGFBR3 high versus negative, low or intermediate tumors in cohort 2.

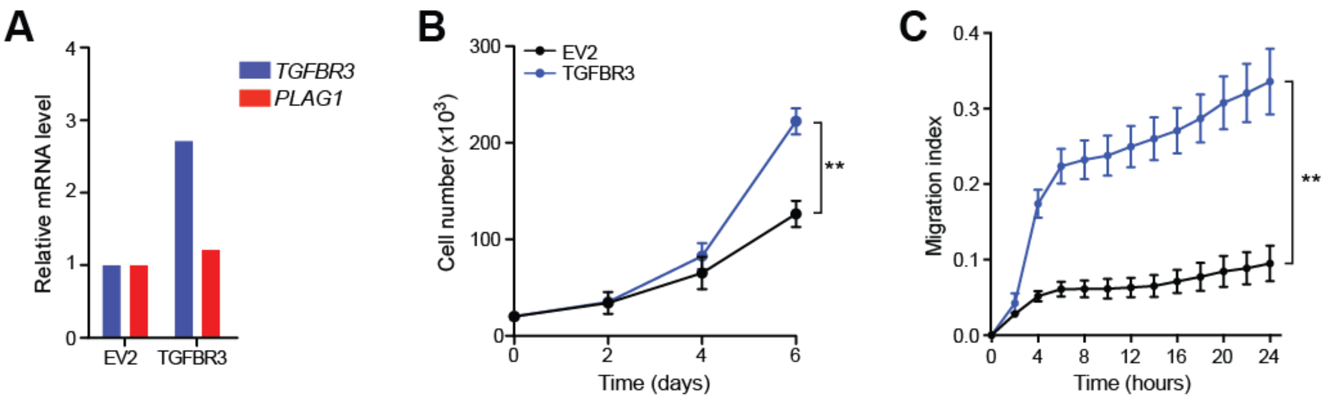

Supplementary Figure 21. Overexpression of *TGFBR3* causes increased proliferation and migration of TCG580 cells, which are defined by a *PLAG1* fusion but normal *TGFBR3*. (A) RT-QPCR showing relative *TGFBR3* and *PLAG1* mRNA levels. Data are mean expression normalized to EV2 levels, using 3 technical replicates/group (B) Proliferation of cells infected with denoted constructs. Data are mean ± SEM, using 3 technical replicates/group. The experiment was replicated 2 times. (C) Migration of cells infected with denoted constructs. Data are mean ± SEM, using 4 technical replicates/group. The experiment was replicated 2 times. **$P < 0.01$; 2-way ANOVA.

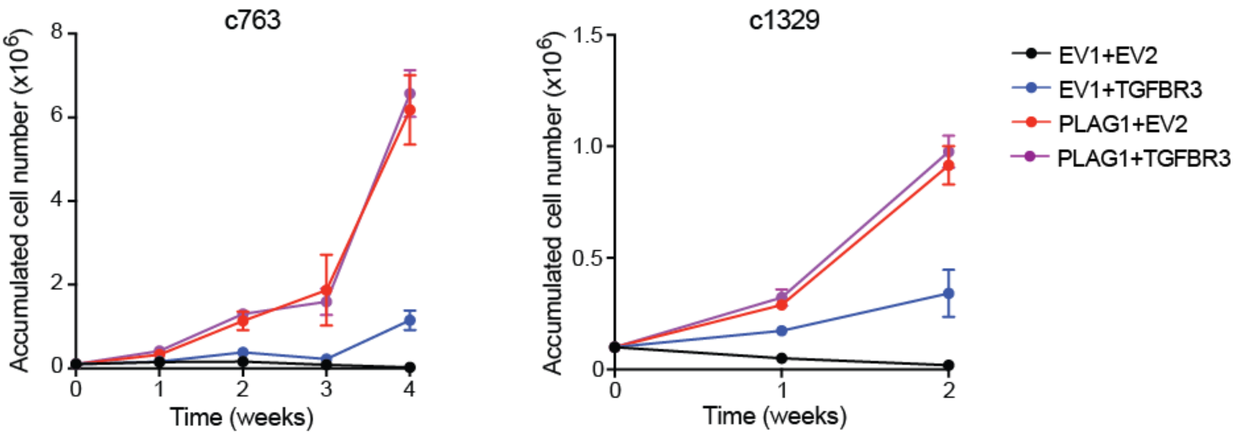

Supplementary Figure 22. Overexpression of *TGFBR3* and/or *PLAG1* causes prolonged survival and growth of primary PA cells. Cells were grown on 6-well plates; 100.000 cells were replated after counting every week. Data are mean ± SEM, using 3 technical replicates per time point, and was adjusted for previous replatings.

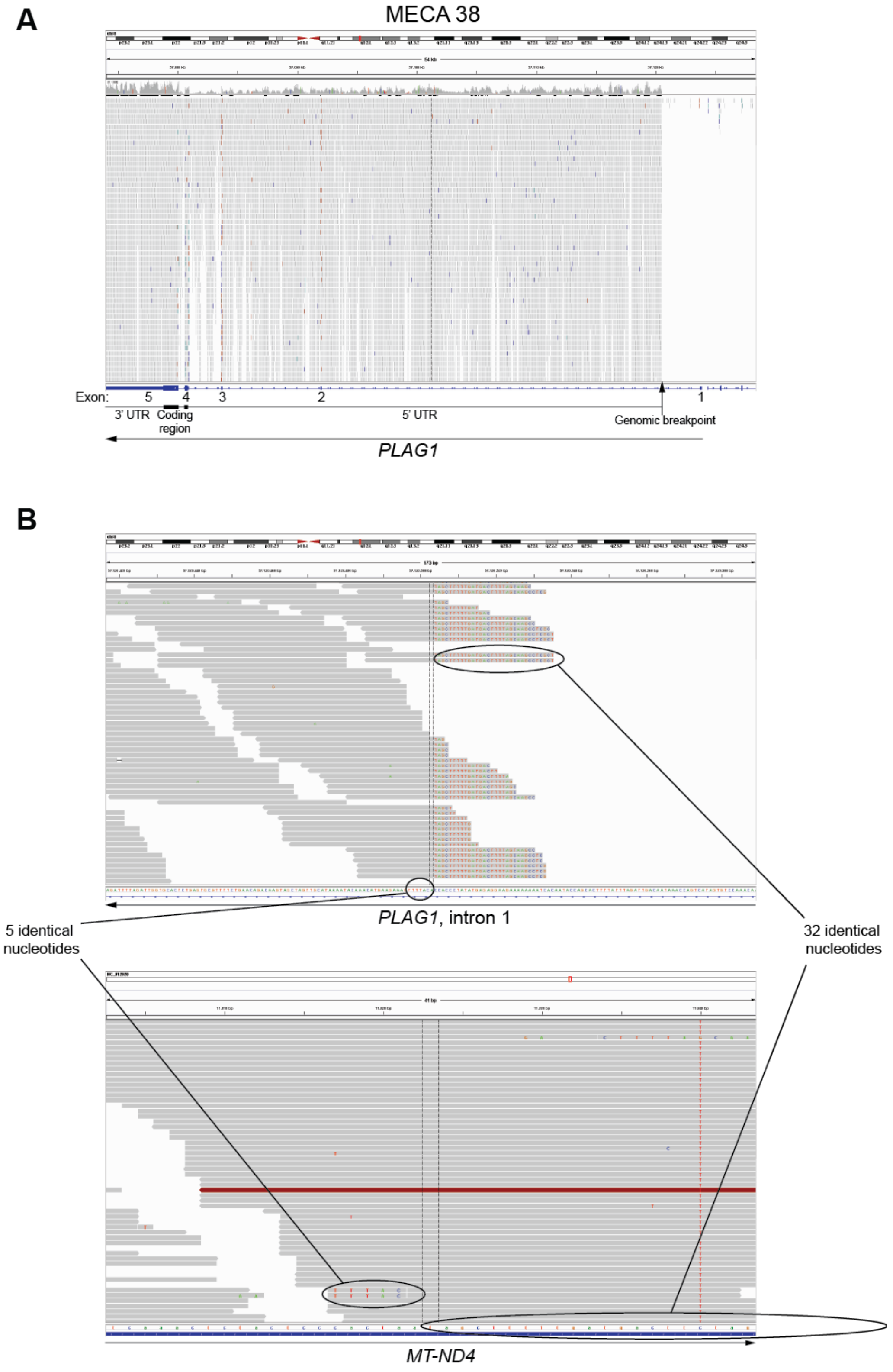

Supplementary Figure 23. Detection of the mitochondrial/nuclear DNA fusion gene *ND4-PLAG1* using RNA sequencing. (A) IGV screenshot showing the genomic breakpoint in intron 1 of *PLAG1*, leading to the formation of a large 5' UTR. (B) Genomic breakpoints in *PLAG1* (top) and *MT-ND4* (bottom), with overlapping nucleotides circled.

A

MECA38

gDNA:
Primers3F+1R
NNNNNCTNNNNNCTATGTAAAACTTTCTTCATGTTTGTATTTTATGCAACTAGCTACTTGTCTGTTCAGAAAACGCA
CTCAGAGTGCACCAATCTAAAATCTGTAATCGCTTAGAGAGTCTTATTTAGATGGGGCACAGCACAATATTTAATTT
GAAGTGATTGTACTGAGCTTTTGTGATGCCTTAGAAATACTACCTGTTGAACATCCTTTTCTGAAGCTATTAAATTA
TACCAGTATACAGAAATAATGTCATGATATTGTGTAGTCAAAATCTTTATTTCAGTTGGAATCNGANAAACCTCAAA
AANNANGAGANNNAGNNNTTNNNNGGGCANCGAGGNTTNNANGTNNTTAAGGTNANGGGTGATNAACCCCCTTGGGG
NTGTGCNNANNNGGNNNNGNNANTTANGGGCTGGANNAATNTTNNNNTCANCCNNGAAAGNACTNNGNGTGNATCCN
GNGGNANTNAACGTATGGTAACTTTCANAACTGANTAANCCGNAGATNATTNANNGGANNNGTTTTGGTCATGTATT
TNGAAGAACACNAANAGGGAANAAAAAAAGTATANCCGGCGAGTACTATCTGAGAGTNGTANNTCTCAAGAGNNNNN
ANATCTATNTTTGTCNNNNCTCATANNANNNNNNCAGGGNNGNTTNGNNNNCNNTNNNNNNCNGNNAACNCGGNN

cDNA
Primers4F+1R
NNNNNNNNNNNNNNNNNGGGTTAGGGTGGTTATAGTAGTGTGCATGGTTATTACTTTTATTTGGAGTTGCACCAAAA
TTCTTGGGGCCTAAGACCAATGGATAGCTGTTATCCTTTAAAAGTTGAGAAAGCCATGTTGTTAGACATGGGGGCAT
GAGTTAGCAGTTCTTGTGAGCTTTCTCGGTAAATAAGGGGTCGTAAGCCTCTGTTGTCAGATTCACAATCTGATGTT
TTGGTTAAACTATATTTACAAGAGGAAAACCCGGTAATGATGTCGGGGTTGAGGGATAGGAGGAGAATGGGGGATAG
GTGTATGAACATGAGGGTGTTTTCTCGTGTGAATGAGGGTTTTATGTTGTTAATGTGGTGGGTGAGTGAGCCCCATT
GTGTTGTGGTAAATATGTAGAGGGAGTATAGGGCTGTGACTAGTATGTTGAGTCCTGTAAGTAGGAGAGTGATATTT
GATCAGGAGAACGTGGTTACTAGCACAGAGAGTTCTCCCAGTAGGTTAATAGTGGGGGGTAAGGCGAGGTTAGCGAG
GCTTGCTAAAAGTCATCAAAAAGCTATGTAAAACTTTCTTCATGTTTGTATTTTATGCAACTAGCTACTTGTCTGTT
CAGAAAACGCACTCAGAGTGCACCAATCTAAAATCTGTAATCGCTTAGAGAGTCTTATTTAGATGGGGCACAGCACA
ATATTTAATTTGAAGTGATTGTACTGAGCTTTTGTGATGCCTTANAAATACTACCTGTTGAACATCCTTTTCTGAAG
TACCAGTATACAGAATATGTCATGATATTGTGTAGTCAAATCTTNATTCANNNNNNNNGGGNNNNNNNNNNNTNNN
NNNNNNNNNNNNAAANNNNNNN

Primers4F+3R
NNNNNNNNNNNNNNNNGGTTAGGGTGGTTATAGTAGTGTGCATGGTTATTACTTTTATTTGGAGTTGCACCAAAATT
CTTGGGGCCTAAGACCAATGGATAGCTGTTATCCTTTAAAAGTTGAGAAAGCCATGTTGTTAGACATGGGGGCATGA
GTTAGCAGTTCTTGTGAGCTTTCTCGGTAAATAAGGGGTCGTAAGCCTCTGTTGTCAGATTCACAATCTGATGTTTT
GGTTAAACTATATTTACAAGAGGAAAACCCGGTAATGATGTCGGGGTTGAGGGATAGGAGGAGAATGGGGGATAGGT
GTATGAACATGAGGGTGTTTTCTCGTGTGAATGAGGGTTTTATGTTGTTAATGTGGTGGGTGAGTGAGCCCCATTGT
GTTGTGGTAAATATGTAGAGGGAGTATAGGGCTGTGACTAGTATGTTGAGTCCTGTAAGTAGGAGAGTGATATTTGA
TCAGGAGAACGTGGTTACTAGCACAGAGAGTTCTCCCAGTAGGTTAATAGTGGGGGGTAAGGCGAGGTTAGCGAGGC
TTGCTAAAAGTCATCAAAAAGCTATGTAAAACTTTCTTCATGTTTGTATTTTATGCAACTAGCTACTTGNNNNNNNA
AAAAAANNNNAANNNNNNNNNNNNNTTNNNTAANNGNNNTCNNNTANNNNTCGTANNNNNAANNNNNNTNNCACCNNN
NNNNNNNNTNNN

B

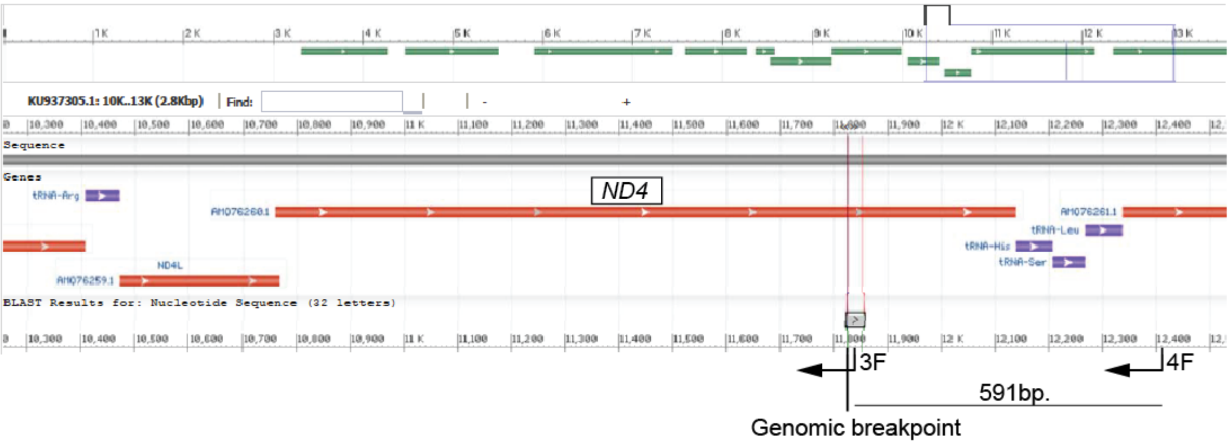

Supplementary Figure 24. Confirmation of *ND4-PLAG1* using Sanger sequencing. (A) Sequences of DNA extracted from PCR products of the denoted primer pairs. Nucleotides that match the *ND4* and *PLAG1* sequences are marked in yellow and blue, respectively. (B) Overview of the ND4 locus and surrounding genes, and locations of primers 3F and 4F.

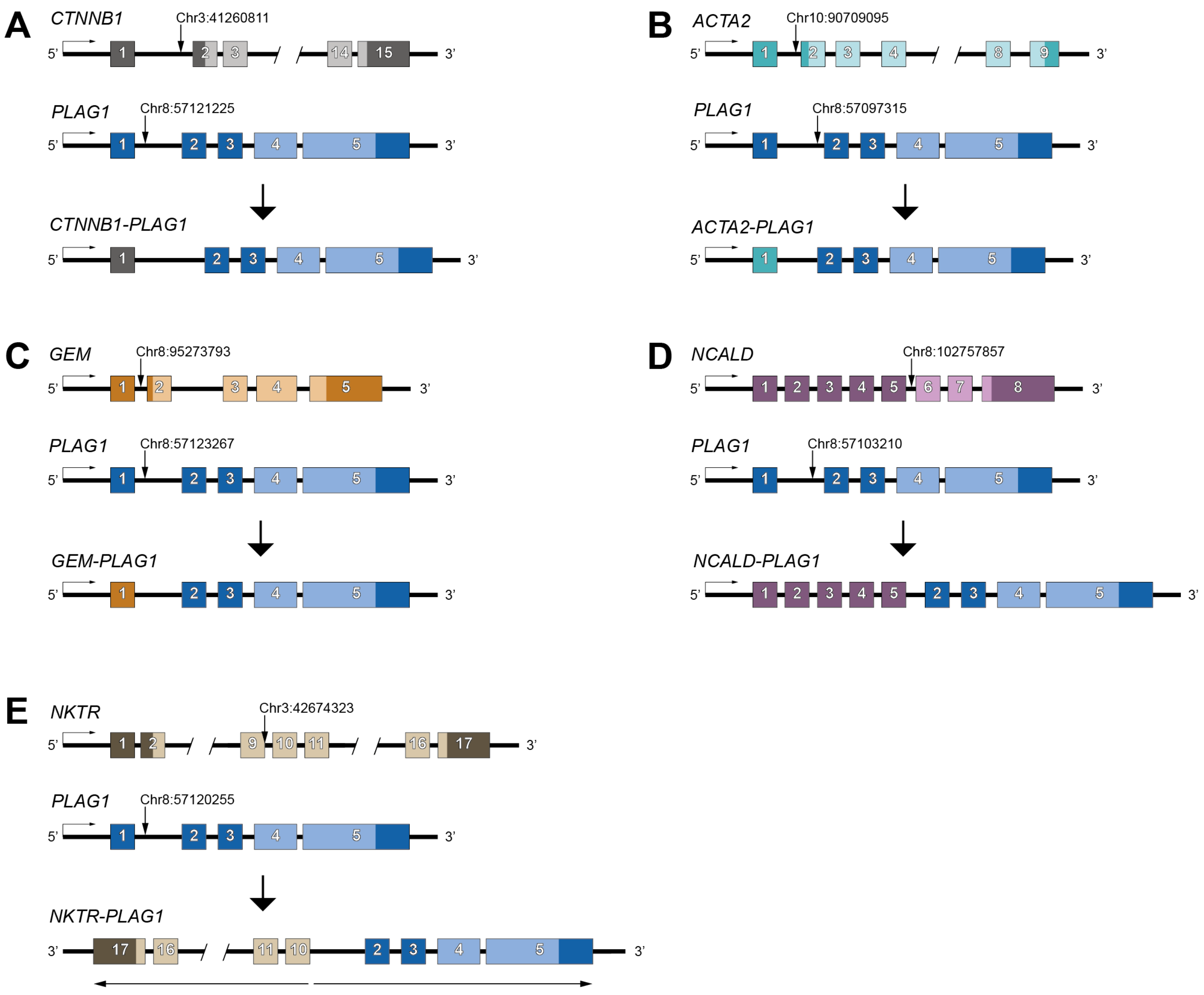

Supplementary Figure 25. Other *PLAG1* fusion genes. Illustrations of the *CTNNB1-PLAG1* (A), *GEM-PLAG1* (B), *ACTA2-PLAG1* (C) and *NCALD-PLAG1* (D) fusion genes, detected in one case each. (E) Illustration of the *NKTR-PLAG1* fusion gene detected in one case, where the inverted intron 9 of *NKTR* is fused with intron 1 of *PLAG1*. Arrows show locations of the genomic breakpoints.

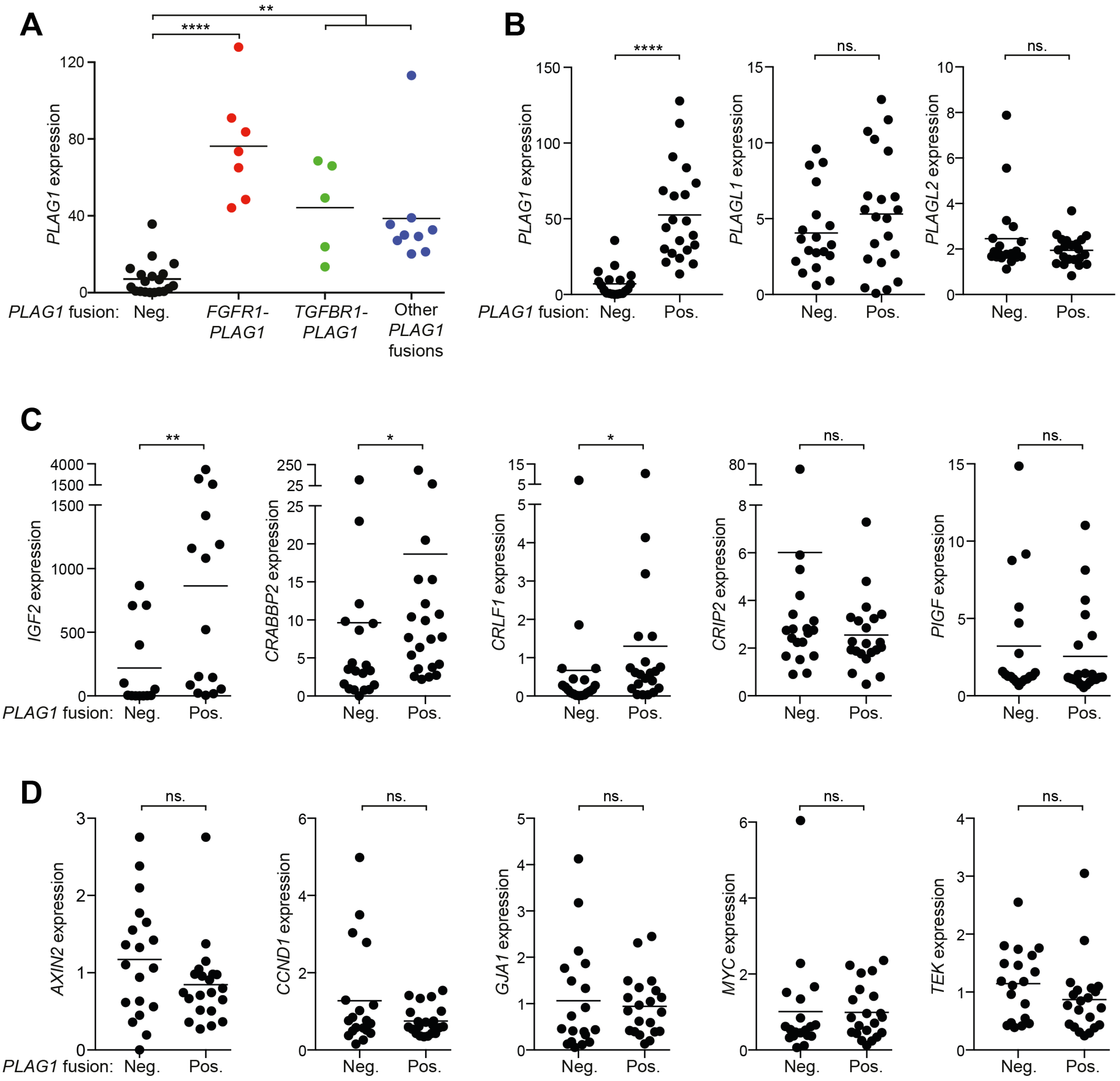

Supplementary Figure 26. Effects of *PLAG1* rearrangements on gene expression. (A) *PLAG1* expression (FPKM) in tumors grouped by *PLAG1* fusion status. $^{**}P < 0.01$, $^{****}P < 0.0001$; 1-way ANOVA with Dunnett's multiple comparison test. (B-D) Expression of (B) *PLAG1* family members, (C) putative *PLAG1* effector genes, and (D) Wnt signaling effector genes, in *PLAG1* fusion negative- and positive cases. $^{*}P < 0.05$, $^{**}P < 0.01$, $^{****}P < 0.0001$; Mann-Whitney test. Horizontal lines represent mean values.

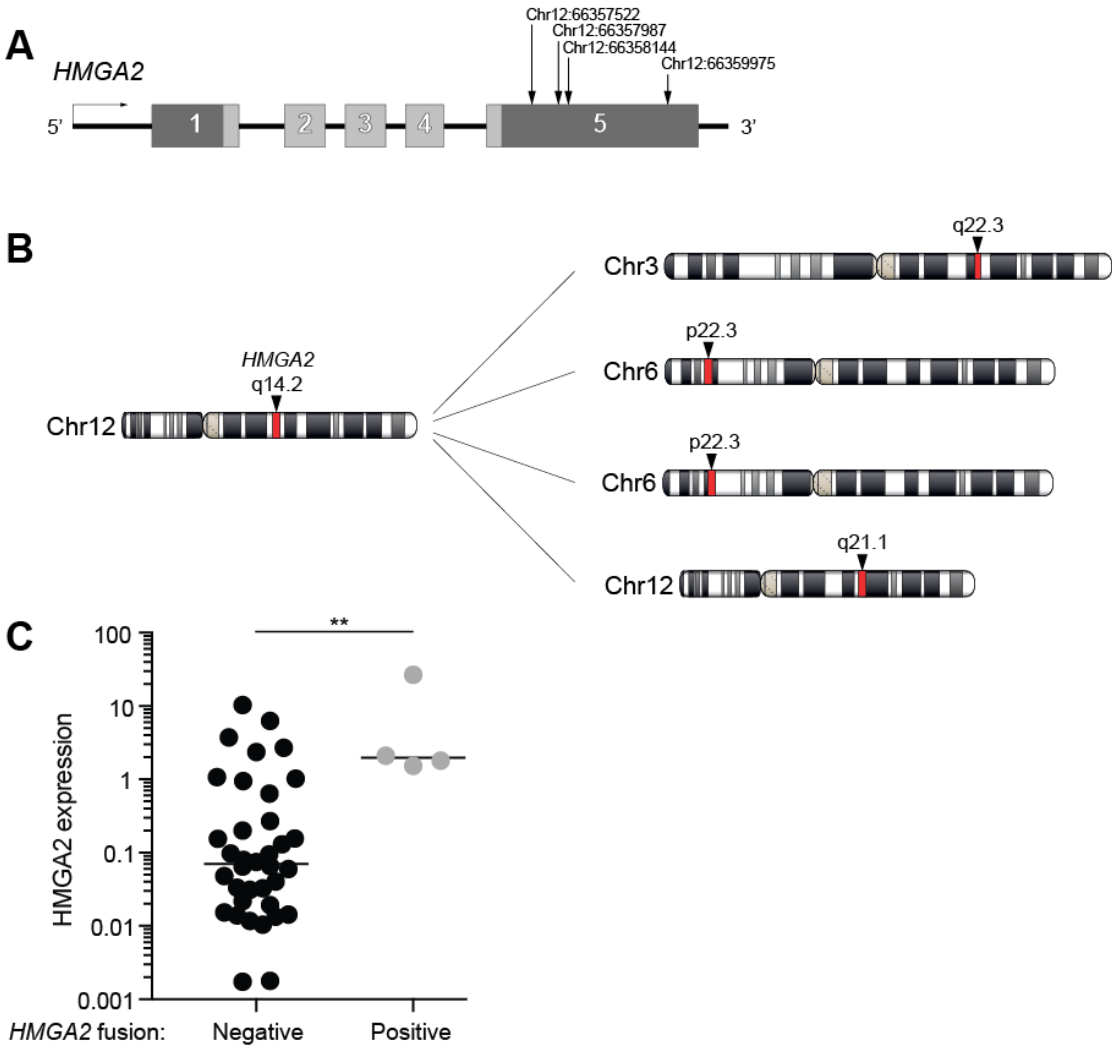

Supplementary Figure 27. *HMGA2* rearrangements. (A) Illustration of *HMGA2* with arrows denoting breakpoint positions in the 4 tumors with *HMGA2* rearrangements. (B) Genomic position of the *HMGA2* gene (left) and of the loci fused with *HMGA2* in the 4 rearrangement-positive tumors (right). (C) *HMGA2* expression (FPKM) based on RNA sequencing in tumors negative or positive for *HMGA2* rearrangements. $^{**}P < 0.01$; Student's t test. $n = 36 + 4$. Horizontal lines represent mean values.

A

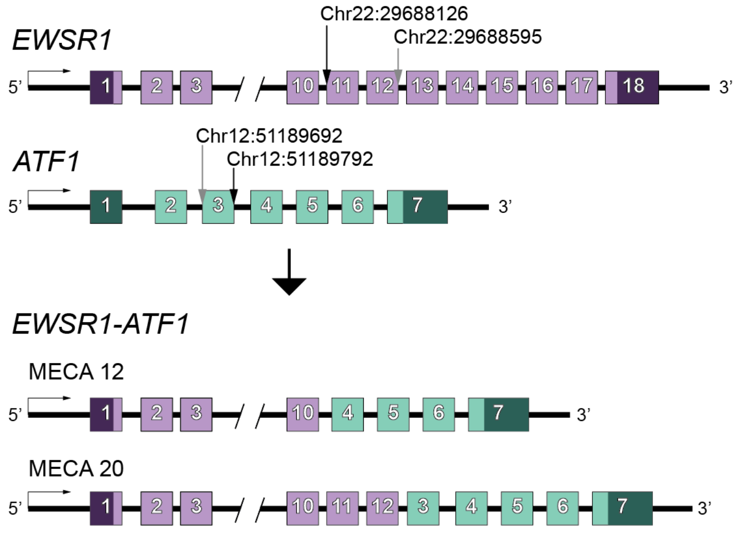

B

MECA 12

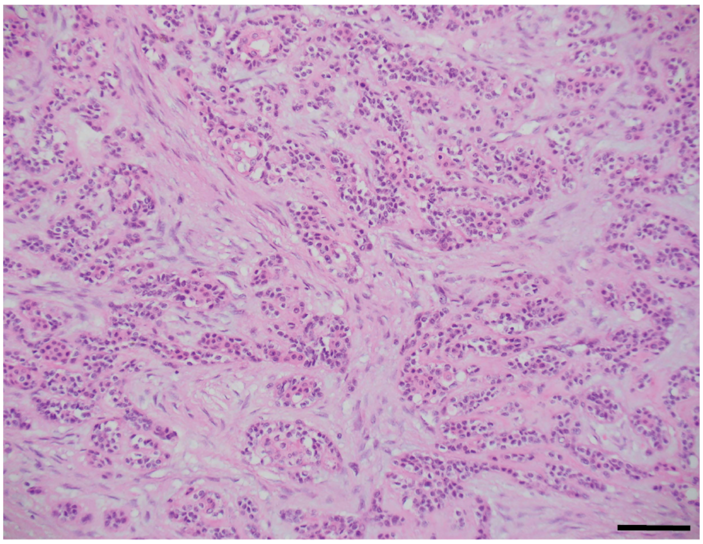

Supplementary Figure 28. The *EWSR1-ATF1* fusion gene. (A) Illustration showing localization of the breakpoints in the two cases of *EWSR1-ATF1*. (B) Example of clear cell features in a MECA with an *EWSR1-ATF1* fusion (H/E staining). Scale bar, 100 μm.

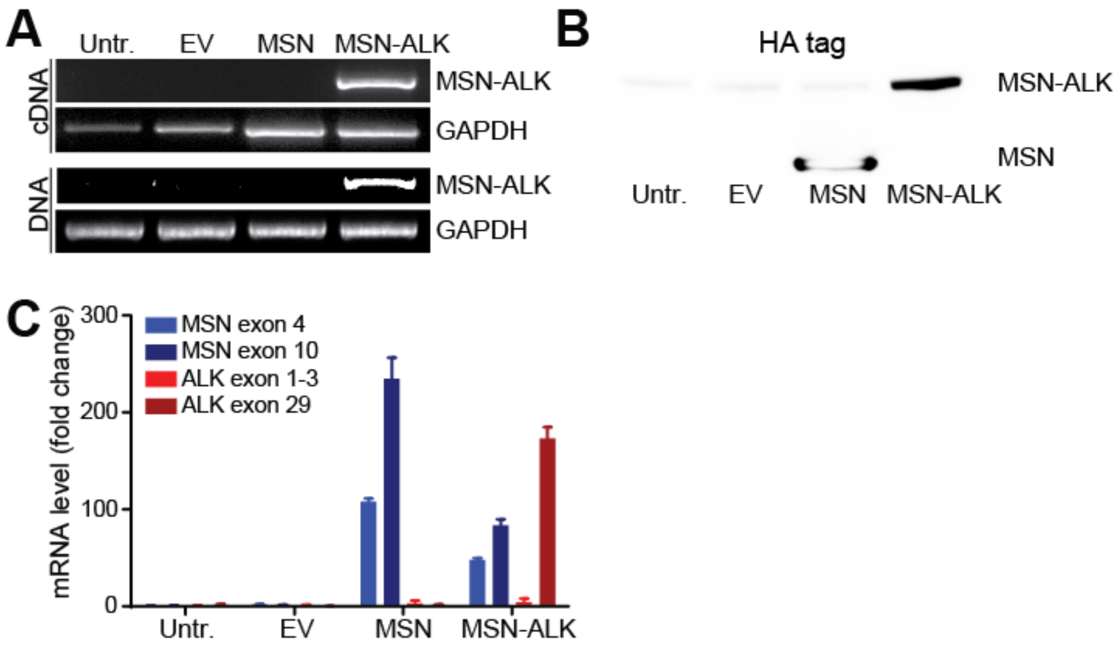

Supplementary Figure 29. Confirmation of MSN and MSN-ALK expression in infected HSG cells. (A) PCR (top) and reverse transcriptase (RT)-PCR (bottom) analyses showing presence and expression of the fusion in HSG cells infected with lentivirus expressing *MSN-ALK*. Untr, untreated. (B) Western blot showing detection of the HA-tag in cells expressing *MSN* and *MSN-ALK*. EV, empty vector. (C) Quantitative RT-PCR showing *MSN* and *ALK* RNA expression upstream (*MSN* exon 4 and *ALK* exon 1-3) and downstream (*MSN* exon 10 and *ALK* exon 29) of the *MSN-ALK* breakpoint. Data are mean ± SEM using 3 technical replicates per group.

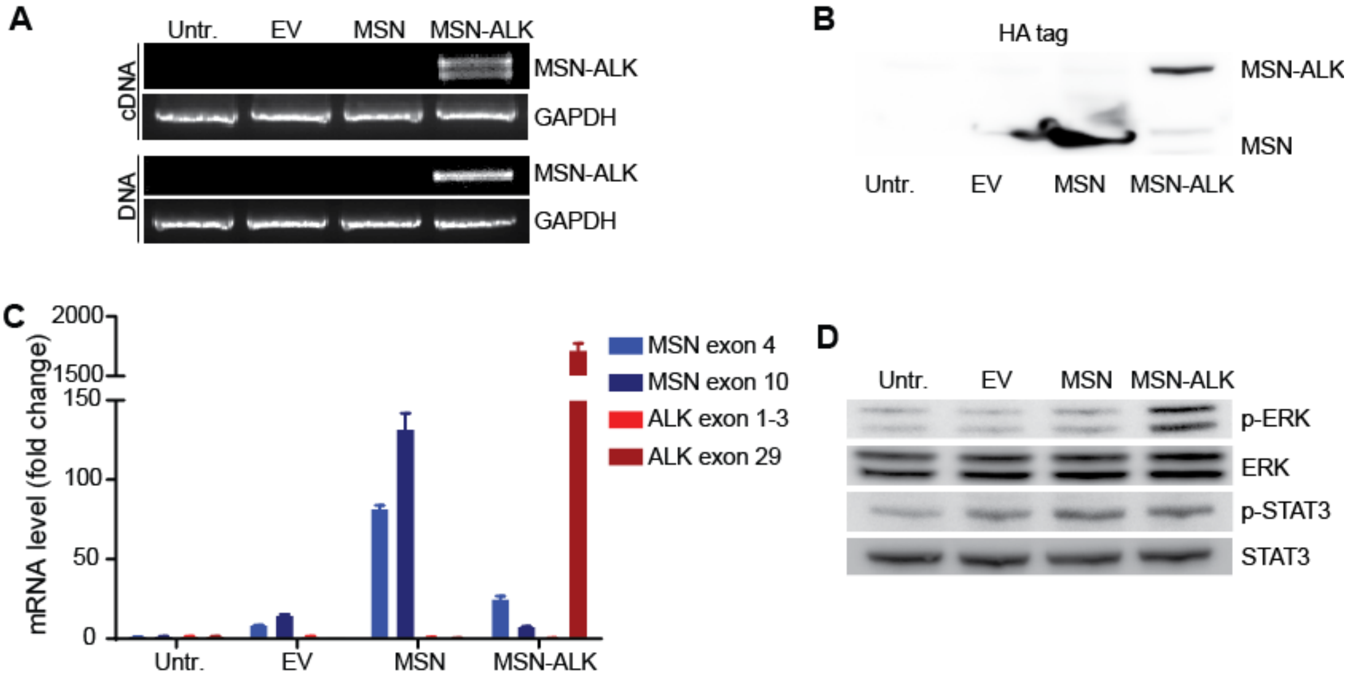

Supplementary Figure 30. *MSN-ALK* expression causes hyperactivation of downstream signaling pathways in HEK cells. (A) PCR (top) and reverse transcriptase (RT)-PCR (bottom) analyses showing presence and expression of the fusion in HEK cells infected with lentivirus expressing *MSN-ALK*. Untr, untreated. (B) Western blot showing detection of the HA-tag in cells expressing *MSN* and *MSN-ALK*. EV, empty vector. (C) Quantitative RT-PCR showing MSN and ALK RNA expression upstream (*MSN* exon 4 and *ALK* exon 1-3) and downstream (*MSN* exon 10 and *ALK* exon 29) of the *MSN-ALK* breakpoint. Data are mean ± SEM using 3 technical replicates per group. (D) Western blot showing levels of ALK downstream signaling proteins in HEK cells expressing empty vector (*EV*), *MSN*, or *MSN-ALK*. P, phosphorylated.

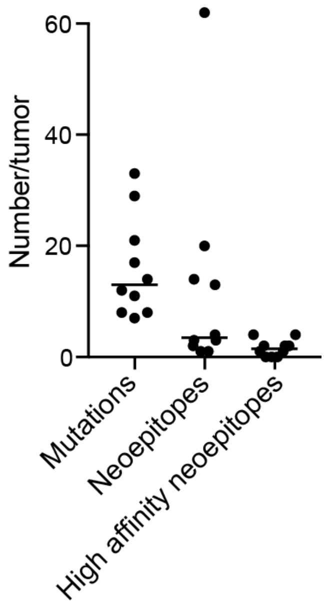

Supplementary Figure 31. Number of mutations and predicted neoepitopes in cohort 1 tumors. Neoepitopes and high affinity neoepitopes are defined as having a predicted binding affinity to MHC molecules of <500nM and <50nM, respectively. Horizontal lines represent median numbers. MECA08 was excluded from the graph, and had 1111 mutations, 1961 neoepitopes, and 163 high affinity neoepitopes.

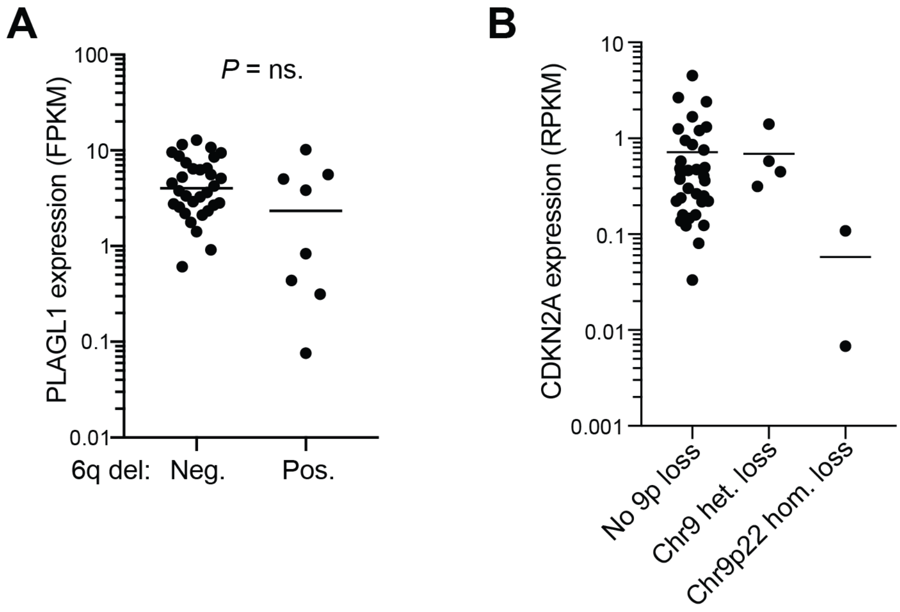

Supplementary Figure 32. Expression of selected genes in tumors with deletions. (A) PLAGL1 expression in 6q deletion positive and negative tumors. (P = 0.15, Mann Whitney test). (B) CDKN2A expression in cases without chromosome 9p deletions, with heterozygous deletion of the entire chromosome 9, and with localized homozygous deletion of 9p22. Horizontal lines show mean values.

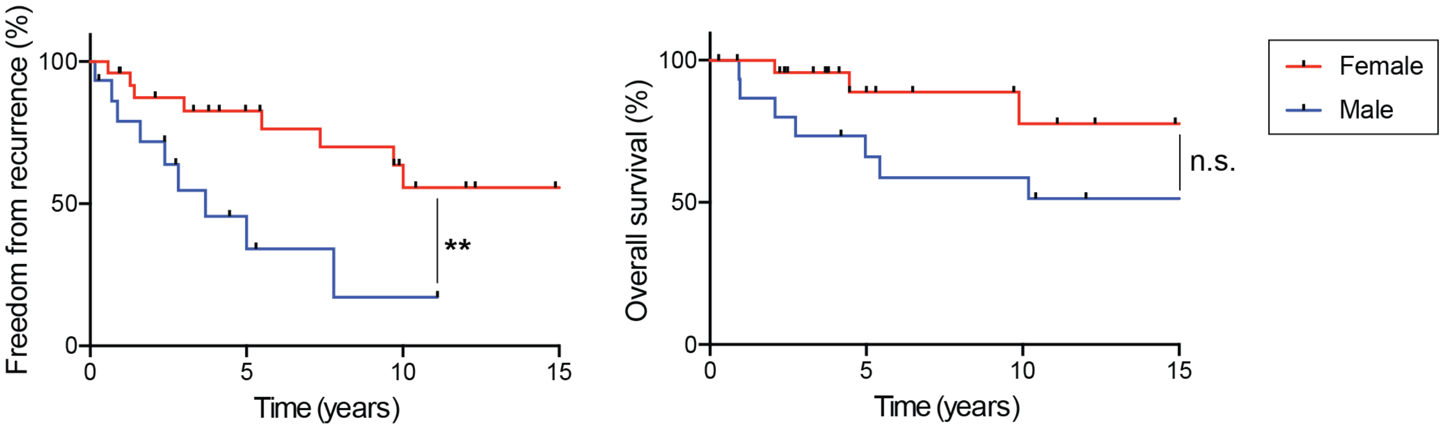

Supplementary Figure 33. Correlation between sex and prognosis. Freedom from recurrence and overall survival in females (*n* = 25) and males (*n* = 15) with MECA. ***P* < 0.01; Log rank test.

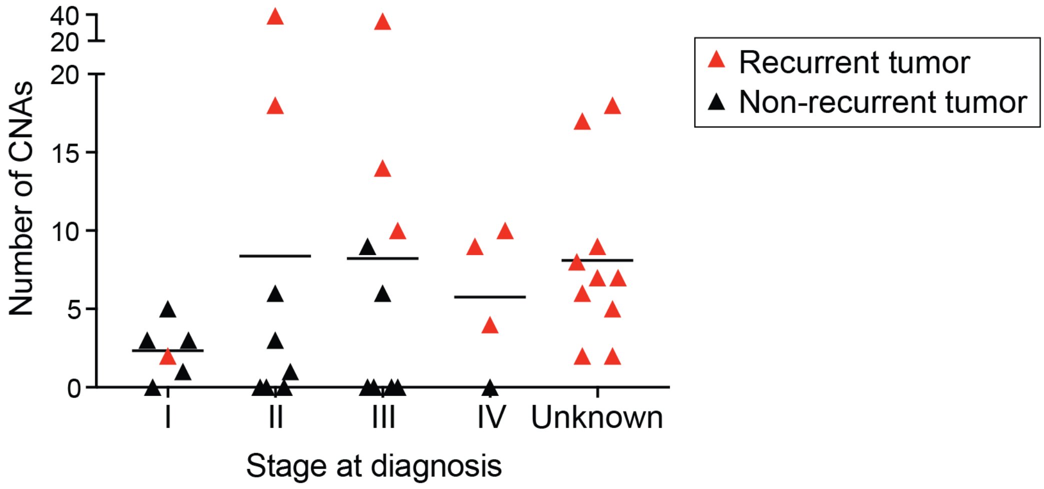

Supplementary Figure 34. Number of CNAs and risk of recurrence in different tumor stages. Stage I, localized disease, tumor ≤2 cm without extraparenchymal extension; stage II, localized disease, tumor 2-4 cm without extraparenchymal extension; stage III, localized disease, tumor >4 cm and/or extraparenchymal extension; stage IV, locally advanced disease (tumor invades the skin, mandible, ear canal, and/or facial nerve) or metastasis to lymph nodes/distant organs.

(Fig. 4B)

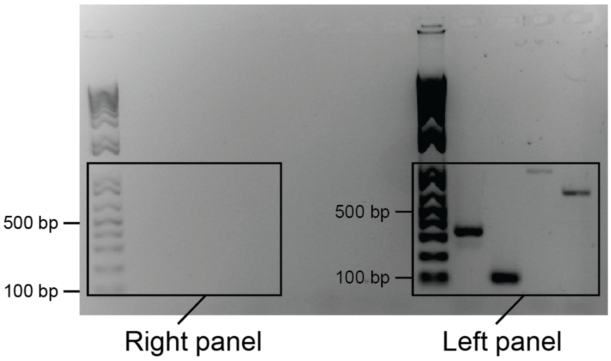

(Fig. 4C)

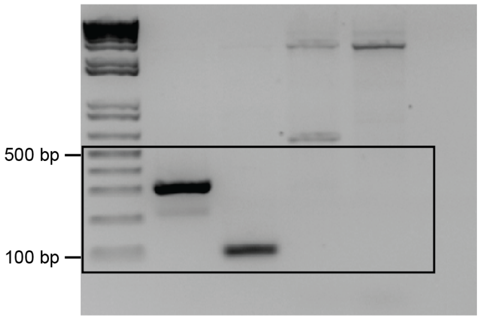

(Fig. 5B)

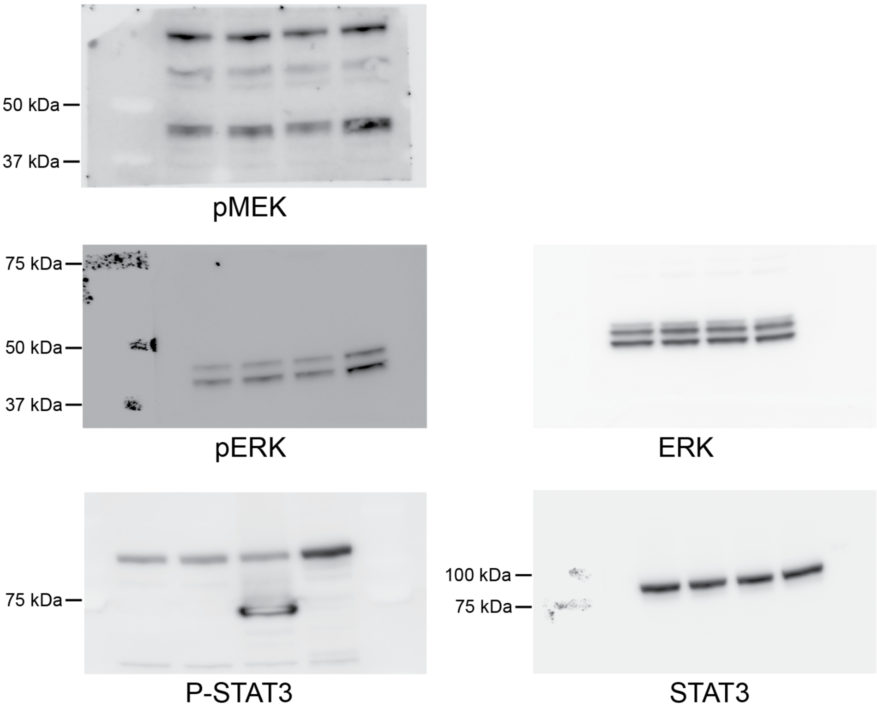

Supplementary Figure 35. Full pictures of PCR gels and western blots that were cropped for inclusion in main figures.

Supplementary Table 1. Clinical information of 40 patients with MECA.

	**n (%) or mean (range)**
**Male sex**	15 (38%)
**Age at diagnosis (y)**	54 (13-90)
**Smoking history**	
Yes	18 (45%)
No	21 (53%)
Unknown	1 (3%)
**Primary tumor site**	
Parotid	33 (83%)
Submandibular	2 (5%)
Minor glands	5 (13%)
**Tumor origin**	
De novo	18 (45%)
Ex-pleomorphic adenoma	22 (55%)
**Disease stage at diagnosis**	
Localized	24 (60%)
Lymph node metastasis	3 (8%)
Distant metastasis	2 (5%)
Unknown	11 (28%)
**Initial treatment**	
Surgery	24 (60%)
Surgery and radiotherapy	16 (40%)
**Disease recurrence**	
No	21 (53%)
Local	10 (25%)
Distant	3 (8%)
Local and distant	6 (15%)
**Status at latest follow-up**	
Alive without disease	25 (63%)
Alive with disease	2 (5%)
Death from disease	9 (23%)
Death from other causes	4 (10%)

Supplementary Table 2. Fusion genes in MECA.

Case	Origin	Fusion gene	5' breakpoint*	3' breakpoint	Detection methods
MECA 01	Ex-PA	-	-	-	-
MECA 02	Ex-PA	*FGFR1-PLAG1*	Chr8:38311736	Chr8:57100178	RNA seq, RT-PCR, FISH
MECA 03	de novo	*TGFBR3-PLAG1*	Chr1:92351435	Chr8:57083748	RNA seq, RT-PCR, FISH
MECA 04	de novo	*NKTR-PLAG1*	Chr3:42674323	Chr8:57120255	RNA seq, FISH
MECA 05	Ex-PA	*FGFR1-PLAG1*	Chr8:38317963	Chr8:57088068	RNA seq, RT-PCR, FISH
MECA 06	Ex-PA	-	-	-	-
MECA 07	Ex-PA	*TGFBR3-PLAG1*	Chr1:92327028	Chr8:57083748	RNA seq, RT-PCR, FISH
MECA 08	de novo	-	-	-	-
MECA 09	de novo	*TGFBR3_PLAG1*	Chr1:92269447	Chr8:57118596	RNA seq, RT-PCR, FISH
MECA 10	de novo	*MSN-ALK*	ChrX:64956787	Chr2:29446394	RNA seq, RT-PCR, FISH
MECA 11	Ex-PA	-	-	-	-
MECA 12	de novo	*EWSR1-ATF1*	Chr22:29688595	Chr12:51189692	
MECA 13	Ex-PA	*FGFR1-PLAG1*	Chr8:38323128	Chr8:57104299	RNA seq, FISH
MECA 14	de novo	*TGFBR3-PLAG1*	Chr1:92327028	Chr8:57083748	
MECA 15	de novo	-	-	-	-
MECA 16	Ex-PA	-	-	-	-
MECA 17	de novo	-	-	-	-
MECA 18	de novo	*TGFBR3-PLAG1*	Chr1:92326040	Chr8:57097807	RNA seq, FISH
MECA 19	de novo	*HMGA2*-Intergenic	Chr12:66358144	Chr6:19395923	RNA seq
MECA 20	de novo	*EWSR1-ATF1*	Chr12:51189792	Chr22:29688126	RNA seq
MECA 21	Ex-PA	*GEM-PLAG1*	Chr8:95273793	Chr8:57123267	RNA seq
MECA 22	Ex-PA	-	-	-	-
MECA 23	Ex-PA	*FGFR1-PLAG1*	Chr8:38322623	Chr8:57119328	RNA seq, FISH
MECA 24	Ex-PA	Unknown-*PLAG1*	-	Chr8:57086934	
MECA 25	de novo	-	-	-	-
MECA 26	Ex-PA	*FGFR1-PLAG1*	Chr8:38317982	Chr8:57102233	RNA seq, FISH
MECA 27	de novo	-	-	-	-
MECA 28	Ex-PA	Unknown-*PLAG1*	-	Chr8:57123798	RNA seq
MECA 29	Ex-PA	*FGFR1-PLAG1*	Chr8:38321934	Chr8:57116115	RNA seq, FISH
MECA 30*	Ex-PA	*FGFR1-PLAG1*	Chr8:38323534	Chr8:57121084	RNA seq, FISH
MECA 31	Ex-PA	Intergenic-*PLAG1*	Chr8:57752529	Chr8:57123041	RNA seq
MECA 32	Ex-PA	*HMGA2*-intergenic	Chr12:66359975	Chr3:139438938	RNA seq
MECA 33	Ex-PA	*ACTA2-PLAG1*	Chr10:90709095	Chr8:57097315	RNA seq
MECA 34	Ex-PA	*HMGA2*-intergenic	Chr12:66357522	Chr12:72598179	RNA seq
MECA 35	de novo	*NCALD-PLAG1*	Chr8:102757857	Chr8:57103210	RNA seq
MECA 36	de novo	-	-	-	-
MECA 37	Ex-PA	*CTNNB1-PLAG1*	Chr3:41260811	Chr8:57121225	RNA seq
MECA 38	Ex-PA	*ND4-PLAG1*	mt:11,823	Chr8:57120503	RNA seq, PCR, RT-PCR
MECA 39	Ex-PA	*HMGA2*-intergenic	Chr6:19534907	Chr12:66357987	RNA seq
MECA 40	Ex-PA	-	-	-	-

*This case also harbored a *TGFBR3-PLAG1* fusion in focal areas, detected by FISH only, see Supplementary Fig. 14B.

Supplementary Table 3. *FGFR1-PLAG1* fusion status in MECA and other salivary gland tumors.

Tumor type	n (total)	n (*FGFR1-PLAG1*)	%	P-value*	Methods	Reference
MECA	40	7	17.5	N/A	RNA seq, RT-PCR, FISH.	Current study
MECA ex-PA	24	7	29.2	N/A	RNA seq, RT-PCR, FISH.	Current study
PA	442	12	2,7	0.0004	Karyotyping, array-CGH, RT-PCR.	[2], Unpubl. (G.S)
ACC	128	0	0	0.00003	WGS, RNA seq, karyotyping.	[2, 3, 4, 5, 6], Unpubl. (L.G.T.M)
MEC	44	0	0	0.004	Karyotyping.	[2]
AC NOS	20	0	0	0.08	Karyotyping.	[2]
WT	20	0	0	0.08	Karyotyping.	[2]
CA ex-PA	18	0	0	0.09	Karyotyping.	[2]
SDC	16	0	0	0.17	RNA seq.	[7]
PLGA	13	0	0	0.17	RNA seq, FISH.	[2, 8], Unpubl. (N.K)
AciCC	12	0	0	0.18	Karyotyping.	[2]
SCC	3	0	0	1.00	Karyotyping.	[2]
BCAC	3	0	0	1.00	FISH, karyotyping.	[2], Unpubl. (N.K)
EMC	2	0	0	1.00	FISH.	Unpubl. (N.K)
SB	1	0	0	1.00	Karyotyping.	[2]
Met PA	1	0	0	1.00	Karyotyping.	[2]

**P*-value testing if the prevalence of *FGFR1-PLAG1* is significantly different between MECA and other tumor types (Fisher's exact test). MECA, myoepithelial carcinoma; PA, pleomorphic adenoma; ACC, adenoid cystic carcinoma; MEC, mucoepidermoid carcinoma; AC NOS, adenocarcinoma not otherwise specified; WT, Warthin's tumor; CA ex-PA, carcinoma ex pleomorphic adenoma; SDC, salivary duct carcinoma; PLGA, polymorphous low-grade adenocarcinoma; AciCC, acinic cell carcinoma; SCC, squamous cell carcinoma; BCAC, basal cell adeno-carcinoma; EMC, Epithelial-myoepithelial carcinoma; SB, sialoblastoma; Met, metastasizing; WGS, whole genome sequencing.

Supplementary Table 4. *TGFBR3-PLAG1* fusion status in MECA and other salivary gland tumors.

Tumor type	n (total)	n (*TGFBR3-PLAG1*)	%	P-value[1]	Methods	References
MECA	40	6	15	N/A	RNA seq, RT-PCR, FISH.	Current study
PA	442	0[2]	0	<0.0001	Karyotyping, array-CGH, RT-PCR.	[2], Unpubl. (G.S)
ACC	128	0	0	0.0001	WGS, RNA seq, karyotyping.	[2, 3, 4, 5, 6], Unpubl. (L.G.T.M)
MEC	44	0	0	0.009	Karyotyping.	[2]
AC NOS	20	0	0	0.17	Karyotyping.	[2]
WT	20	0	0	0.17	Karyotyping.	[2]
CA ex-PA	18	0	0	0.17	Karyotyping.	[2]
SDC	16	0	0	0.17	RNA seq.	[7]
PLGA	13	0	0	0.32	RNA seq, FISH.	[2, 8], Unpubl. (N.K)
AciCC	12	0	0	0.32	Karyotyping.	[2]
SCC	3	0	0	1.00	Karyotyping.	[2]
BCAC	3	0	0	1.00	FISH, karyotyping.	[2], Unpubl. (N.K)
EMC	2	0	0	1.00	FISH.	Unpubl. (N.K)
SB	1	0	0	1.00	Karyotyping.	[2]
Met PA	1	0	0	1.00	Karyotyping.	[2]

[1]*P*-value testing if the prevalence of *TGFBR3-PLAG1* is significantly different between MECA and other tumor types (Fisher's exact test). [2]In 1 of the 442 PAs, karyotyping showed a t(1;8) translocation; this tumor was negative for *TGFBR3-PLAG1* using RT-PCR. MECA, myoepithelial carcinoma; PA, pleomorphic adenoma; ACC, adenoid cystic carcinoma; MEC, mucoepodermoid carcinoma; AC NOS, adenocarcinoma, not otherwise specified; WT, Warthin tumor; CA ex-PA, carcinoma ex-pleomorphic adenoma; SDC, salivary duct carcinoma; PLGA, polymorphous low-grade adenocarcinoma; AciCC, acinic cell carcinoma; SCC, squamous cell carcinoma; BCAC, basal cell adenocarcinoma; EMC, epithelial-myoepithelial carcinoma; SB, sialoblastoma; Met, metastasizing; WGS, whole genome sequencing.

Supplementary Table 5. Predicted HLA-binding neoantigen formation for fusion genes in cohort 1.

Case	Fusion gene	5' breakpoint	3' breakpoint	Neo-epitope sequence[1]	Neo-epitope affinity (nM)[2]	HLA allele
MECA 02	*FGFR1-PLAG1*	8:38311736	8:57100178	-	N/A	N/A
MECA 03	*TGFBR3-PLAG1*	1:92351435	8:57083748	-	N/A	N/A
		1:92327027		SSCLATADWPK	68	HLA-A11:01
MECA 04	*NKTR-PLAG1*	3:42674323	8:57120255	-	N/A	N/A
MECA 05	*FGFR1-PLAG1*	8:38317963	8:57088068	-	N/A	N/A
MECA 07	*TGFBR3-PLAG1*	1:92327028	8:57083748	CLATADWPK	88	HLA-A68:01
				CLATADWPK	411	HLA-A02:16
				LMSSCLATAD	98	HLA-A02:02
				LMSSCLATAD	118	HLA-A02:03
				SSCLATADW	31	HLA-B15:17
			8:57092072	ATAGCLLVL	65	HLA-A02:06
				ATAGCLLVL	67	HLA-B15:17
				CLATAGCLL	19	HLA-A02:02
				CLATAGCLL	92	HLA-A02:16
				CLATAGCLL	107	HLA-A02:12
				CLATAGCLLV	28	HLA-A02:03
				CLATAGCLLV	155	HLA-A02:01
MECA 09	*TGFBR3-PLAG1*	1:92269447	8:57118596	-	N/A	N/A
		1:92327027	8:57083748	LATADWPKW	5	HLA-B58:01
				LATADWPKW	25	HLA-B53:01
				LATADWPKW	74	HLA-B57:03
				MSSCLATADW	18	HLA-B57:01
			8:57092072	MSSCLATAG	421	HLA-B35:01
				TAGCLLVLPW	254	HLA-B58:01
				TAGCLLVLPW	259	HLA-B57:01
MECA 10	*MSN-ALK*	x:64956787	2:29446394	KAQQVYRRK	135	HLA-A30:01
				QTKKAQQVY	405	HLA-A30:02
				QVYRRKHQEL	170	HLA-B08:01
MECA 12	*EWSR1-ATF1*	22:29688595	12:51189692	-	N/A	N/A
	ATF1-EWSR1	12:51189692	22:29688595	ISHIAQQMNK	94	HLA-A03:01

[1]An 8-11 amino acid sequence is shown, depending on coding frame; the fusion junction is in the middle of the sequence. [2]Neoantigens with epitope affinity <50nM are considered strong binders.

Supplementary Table 6. Significant CNAs in MECA de novo and MECA ex-PA tumors.

Chromosome region	Alteration	MECA (n=37)	MECA de novo (n=15)	MECA Ex-PA (n=22)	P-value**
1pcen-p32.3	Loss	6 (16%)	0 (0%)	6 (27%)	ns.
1qcen-qter	Gain	11 (30%)	1 (6.7%)	10 (45%)	0.014
5pter-pcen	Gain	7 (19%)	3 (20%)	4 (18%)	ns.
6qcen-qter	Loss	8 (22%)	0 (0%)	8 (36%)	0.012
8*	Gain	16 (43%)	2 (13%)	14 (64%)	0.003
11pter-pcen	Loss	7 (19%)	1 (6.7%)	6 (27%)	ns.
16qcen-qter	Gain	7 (19%)	2 (13%)	5 (23%)	ns.

* Gain of whole chromosome 8 or complex rearrangements (including ring formation).
**Fisher's exact test.

Supplementary Table 7. Clinico-pathological characteristics and copy number alterations in 17 pleomorphic adenomas profiled by arrayCGH.

Case No.	Diagnosis	Sex/Age	Location	Size (cm)	CNAs
1	PA	K/68	PG	2	No
2	PA	K/72	PG	2.5	No
3	PA	K/36	PG	1.5	No
4	PA	K/48	PG	3	No
5	PA	K/59	PG	2.5	No
6	PA	K/39	PG	1.5	No
7	PA	K/48	PG	1.8	No
8	PA	K/52	PG	1.3	No
9	PA	K/74	PG	1.5	No
10	PA	M/31	PG	2.5	No
11	PA	M/54	PG	2	No
12	PA	M/82	PG	2.2	No
13	PA	M/48	PG	1.9	No
14	PA	M/36	PG	3.5	Gain 16p13.11-p12.3 (3.4 Mb, 62 genes)
15	PA	M/21	PG	1.5	No
16	PA	K/20	PG	1.3	No
17	PA	M/54	PG	2.6	No

PA, pleomorphic adenoma; PG, parotid gland.

Supplementary Table 8. Primer sequences.

**Primer name**	**Primer sequence (5'-3')**	**Nucleic acid**
TGFBR3_PLAG1_F	CTCTTCCCAGCGAGTGAAGG	cDNA
TGFBR3_PLAG1_R	CATGCAAGGCCAAGTGACG	cDNA
FGFR1_PLAG1_F1	AGGATCGAGCTCACTGTGGA	cDNA
FGFR1_PLAG1_R1	TCCCATTGACTCTTCGTGGA	cDNA
FGFR1_PLAG1_F2	AGGATCGAGCTCACTGTGGA	cDNA
FGFR1_PLAG1_R2	TCCCATTGACTCTTCGTGGA	cDNA
MSN_ALK_F	ACAAGCGGATCTTGGCCTTG	cDNA/DNA
MSN_ALK_R	CGGACACCTGGCCTTCATAC	cDNA/DNA
EWSR1_ATF1_F	TTGATCGTGGAGGCATGAGC	cDNA
EWSR1_ATF1_R	TTGCCAACTGTAAGGCTCCA	cDNA
PLAG1_ND4_1F	AGAACGTGGTTACTAGCACAGAG	cDNA/DNA
PLAG1_ND4_1R	TGAGGTTTCTCCGATTCCAAC	cDNA/DNA
PLAG1_ND4_2F	TTTGATCAGGAGAACGTGGTTA	cDNA/DNA
PLAG1_ND4_2R	TTAAATATTGTGCTGTGCCCCATC	cDNA/DNA
PLAG1_ND4_3F	GGGTAAGGCGAGGTTAGCGA	cDNA/DNA
PLAG1_ND4_3R	GCGTTTTCTGAACAGACAAGTAGC	cDNA/DNA
PLAG1_ND4_4F	GGGTTAACGAGGGTGGTAAGG	cDNA/DNA
PLAG1_ND4_5F	GGTATGGTTTGAGTAGTCCTCC	cDNA/DNA
PLAG1_ND4_6F	AGTAGGGTTAGGATGAGTGGGA	cDNA/DNA
GAPDH_F	GTCAAGGCTGAGAACGGGAA	cDNA/DNA
GAPDH_R	CCGTTCAGCTCAGGGATGAC	cDNA/DNA
**For quantitative PCR**		
**Name**	**Primer sequence (5'-3')**	**Nucleic acid**
MSN_exon4_F	CGTGCCAAGTTCTACCCTGA	cDNA
MSN_exon4_R	GTCTCAGGCGGGCAGTAAAT	cDNA
MSN_exon10_F	GGAACTTGAGCAGGAACGGA	cDNA
MSN_exon10_R	GCCAGCTGTTCCTGAGTCTT	cDNA
ALK_exon1-3_F	TCCAGATCTTCGGGACTGGT	cDNA
ALK_exon1-3_R	ACCATATCGGCTGCGATGAG	cDNA
ALK_exon29_F	CAACACCGCTTTGCCGATAG	cDNA
ALK_exon29_R	TCCCGTTTTGCCTGTTGAGA	cDNA
STLM_F	AAGCCAAAAGATGGGCAGGA	cDNA
STLM_R	GCTTCCTTCTCGACAGGGTC	cDNA

Supplementary Table 9. FISH probes.

Clone	Gene/Locus	Band	Color
***TGFBR3-PLAG1* (2-color fusion probe)**			
RP11-135F24	3'TGFBR3	1p22.2-1p22.1	Red
RP11-43G6	3'TGFBR3		
RP11-77A10	5'TGFBR3		
RP11-28O2	5'TGFBR3		
RP11-92A9	5'PLAG1	8q12.1	Green
RP11-111I18	5'PLAG1		
RP11-246A9	3'PLAG1		
***MSN-ALK* (2-color fusion probe)**			
RP3-323B6	5'MSN	Xq12	Red
RP11-368D24	MSN		
RP11-434B14	3'MSN		
RP11-484N8	3'MSN		
CTD-3049L15	3'ALK	2p23	Green
RP11-100C1	3'ALK		
RP11-701P18	5'ALK		
RP11-644H8	5'ALK		
***NKTR-PLAG1* (2-color fusion probe)**			
RP11-776E8	5'NKTR	3p22.1	Red
RP11-219I21	5'NKTR		
RP11-13K13	3'NKTR		
RP11-141M3	3'NKTR		
RP11-92A9	5'PLAG1	8q12.1	Green
RP11-111I18	5'PLAG1		
RP11-246A9	3'PLAG1		
***NKTR-3'PLAG1-5'PLAG1* (3-color fusion probe)**			
RP11-776E8	5'NKTR	3p22.1	Green
RP11-219I21	5'NKTR		
RP11-13K13	3'NKTR		
RP11-141M3	3'NKTR		
RP11-92A9	5'PLAG1	8q12.1	Orange
RP11-111I18	5'PLAG1		
RP11-246A9	3'PLAG1		Red
***TGFBR3-PLAG1-FGFR1* (3-color fusion probe)**			
RP11-135F24	3'TGFBR3	1p22.2-1p22.1	Red
RP11-43G6	3'TGFBR3		
RP11-77A10	5'TGFBR3		
RP11-28O2	5'TGFBR3		
RP11-92A9	5'PLAG1	8q12.1	Green
RP11-111I18	5'PLAG1		
RP11-246A9	3'PLAG1		
RP11-350N15	FGFR1	8p11	Orange

References, Supplementary information

1. Ben Lassoued A, Nivaggioni V, Gabert J. Minimal residual disease testing in hematologic malignancies and solid cancer. *Expert review of molecular diagnostics* **14**, 699-712 (2014).

2. Mitelman F JBaMF. Mitelman Database of Chromosome Aberrations and Gene Fusions in Cancer (2016). (ed^(eds).

3. Rettig EM*, et al.* Whole-Genome Sequencing of Salivary Gland Adenoid Cystic Carcinoma. *Cancer prevention research (Philadelphia, Pa)* **9**, 265-274 (2016).

4. Mitani Y*, et al.* Novel MYBL1 Gene Rearrangements with Recurrent MYBL1-NFIB Fusions in Salivary Adenoid Cystic Carcinomas Lacking t(6;9) Translocations. *Clinical cancer research : an official journal of the American Association for Cancer Research* **22**, 725-733 (2016).

5. Brayer KJ, Frerich CA, Kang H, Ness SA. Recurrent Fusions in MYB and MYBL1 Define a Common, Transcription Factor-Driven Oncogenic Pathway in Salivary Gland Adenoid Cystic Carcinoma. *Cancer discovery* **6**, 176-187 (2016).

6. Ho AS*, et al.* The mutational landscape of adenoid cystic carcinoma. *Nature genetics* **45**, 791-798 (2013).

7. Dalin MG*, et al.* Comprehensive Molecular Characterization of Salivary Duct Carcinoma Reveals Actionable Targets and Similarity to Apocrine Breast Cancer. *Clinical cancer research : an official journal of the American Association for Cancer Research* **22**, 4623-4633 (2016).

8. Weinreb I*, et al.* Hotspot activating PRKD1 somatic mutations in polymorphous low-grade adenocarcinomas of the salivary glands. *Nature genetics* **46**, 1166-1169 (2014).
