# Supplementary material for: Multi-dimensional genomic analysis of myoepithelial carcinoma identifies prevalent oncogenic gene fusions
Source: Nat Commun. 2017 Oct 30;8:1197. doi: 10.1038/s41467-017-01178-z (PMC5662567; doi:10.1038/s41467-017-01178-z)
Supplement: Supplementary file 3 — Description of Additional Supplementary Files [file 41467_2017_1178_MOESM3_ESM.pdf]

Description of Additional Supplementary Files

File Name: Supplementary Data 1
Description: All somatic mutations in cohort 1

File Name: Supplementary Data 2
Description: Somatic mutations in cancer-related genes, cohort 2

File Name: Supplementary Data 3
Description: Copy number alterations
